# Psychotomimetic Cannabinoid Hyperlocomotion Involves GSK-3β Signaling at Striatopallidal Neurons in Male Mice

**DOI:** 10.1016/j.bpsgos.2026.100816

**Published:** 2026-08-28

**Authors:** Giovanni Tomaselli, Luigi Bellocchio, Pierre-Louis Raux, Valérie Roullot-Lacarrière, Arnau Busquets-Garcia, Valérie Lalanne, Yamuna Mariani, Adeline Cathala, Marion Zanese, Astrid Cannich, Agnès Grel, Doriane Gisquet, Miguel Mondesir, Mathilde Metna-Laurent, Guillaume Drutel, Giovanni Marsicano, Pier Vincenzo Piazza, Jean-Michel Revest, Monique Vallée

**Affiliations:** a University of Bordeaux, French National Institute of Health and Medical Research, Neurocentre Magendie, U1215, Bordeaux, France; b Aelis Farma, Bordeaux, France

**Keywords:** AEF0117, D2 striatopallidal neurons, GSK-3β, Pregnenolone, Psychotic-like disorder, THC-induced hyperlocomotion

## Abstract

**Background:**

Hyperlocomotion induced by psychotomimetic agents is considered a proxy for positive psychotic-like symptoms and is used in antipsychotic drug screenings with rodents. Cannabinoids are psychotomimetic drugs in humans, and low doses of cannabinoid CB_1_ receptor (CB1R) agonists induce hyperlocomotion in rodents. However, the cellular and molecular mechanisms underlying this effect remain unexplored so far.

**Methods:**

We used a combination of pharmacological and genetic manipulations in cultured cells and adult male mouse models, together with an intracellular signaling detection method. Our multidisciplinary approach included locomotor activity (*n* = 10–12 mice/group), alphaLISA technology (*n* = 4–12 mice/group), transfection of the β-arrestin 1 protein in STH*dh*^Q7/Q7^ cells (*n* = 4–6/group) and a viral strategy in transgenic lines to selectively block GSK-3β (glycogen synthase kinase-3β) phosphorylation (*n* = 8–10 mice/group). We also used fluorescence in situ hybridization, real-time quantitative and droplet digital polymerase chain reaction, and tandem mass spectrometry.

**Results:**

We showed that the hyperlocomotion induced by a low dose (0.3 mg/kg) of cannabinoid Δ^9^-tetrahydrocannabinol depends on the activation of plasma membrane CB1R located on dopamine D_2_ receptor–positive striatopallidal projecting neurons. This activation regulates the β-arrestin 1/Akt/GSK-3β signaling pathway, which is required for the hyperlocomotor effect. The signaling-specific CB1R inhibitors, pregnenolone and its clinically developed analog, AEF0117, prevent GSK-3β–dependent psychomotor stimulation induced by THC.

**Conclusions:**

This work highlights an intracellular mechanism of CB1R expressed by a selective key neuronal pathway mediating the psychotomimetic effect of THC and, by extension, low-dose cannabis consumption. This discovery will likely aid the development of unprecedented therapeutic modalities for the treatment of psychotic conditions.

## Plain Language Summary

Studies have shown a link between acute psychotic-like symptoms and exposure to cannabis or its main psychoactive component, Δ^9^-tetrahydrocannabinol (THC), in both humans and animal models. Here, we demonstrate the cellular and molecular mechanisms underlying THC-induced psychotomimetic hyperlocomotion in male mice. These mechanisms involve cannabinoid CB_1_ receptor (CB1R)–mediated, β-arrestin 1–dependent inhibition of GSK-3β signaling activity in the cerebello–basal ganglia pathway. Additionally, we demonstrate that the signaling-specific CB1R inhibitors, pregnenolone and its clinically developed analog AEF0117, prevent GSK-3β–dependent psychomotor stimulation induced by THC. These findings shed light on new therapeutic approaches for psychotic disorders associated with cannabinoids.

*Cannabis sativa* is one of the most commonly abused drugs in Europe and worldwide, increasing the risk of cannabis use disorder (1) and related psychosis-like conditions (2, 3, 4, 5, 6, 7). Both cannabis and its main psychoactive component, Δ^9^-tetrahydrocannabinol (THC), have been shown to induce acute psychotic symptoms in humans (6,8, 9, 10) and mirror the addictive and toxic effects of cannabis use in animals (11), including THC-induced acute psychotic-like states (CIAPS) (12). THC exerts dose-dependent biphasic effects on locomotion, food intake, cognition, and reward (12, 13, 14, 15). Specifically, high doses decrease locomotion, while low doses (<0.5 mg/kg) stimulate it in rodents (12,15,16). Cannabinoid CB_1_ receptors (CB1Rs) mediate the psychoactive effects of THC in the brain, including hyperlocomotion (15,17); however, the specific downstream cellular processes remain to be fully elucidated.

CB1Rs are high-abundance 7-transmembrane GPCRs (G protein–coupled receptors) in the mammalian brain. Upon activation by exogenous (e.g., THC) or endogenous agonists (endocannabinoids), CB1R activates a variety of intracellular signaling cascades that are mediated by G proteins but also by β-arrestins. Hyperactivity of CB1R-mediated signaling has been associated with several pathological states. Despite the therapeutic potential of inhibiting CB1R, orthosteric antagonists induce side effects that limit their use (18, 19, 20). To bypass these limitations, attention has shifted to CB1R signaling-specific inhibitors (CB1-SSis), like the endogenous neurosteroid pregnenolone (PREG) and its clinically developed analog AEF0117 (11,21). Rather than altering agonist binding or G protein/cAMP (cyclic adenosine monophosphate) pathways, CB1-SSi selectively block cannabinoid-induced ERK^1/^^2^/MAPK (extracellular signal-regulated kinase 1/2/mitogen-activated protein kinase) activation (11,21). This biased inhibition avoids antagonist-associated side effects while effectively blocking THC-induced behaviors, including CIAPS, across rodents, nonhuman primates, and humans (12,21). This pathway-specific modulation highlights the distinct physiological roles of independent GPCR cascades, an area that remains poorly understood.

Here, using genetic mouse models combined with pharmacological, viral, and molecular tools, we investigated the specific intracellular pathways and cellular localizations that drive low-dose THC-induced psychotomimetic locomotor hyperactivity.

## Methods and Materials

Detailed methods are included in the Supplement and the Key Resources Table.

### Animals

All experiments were conducted on adult male mice (9–10 weeks old at the start), during the light phase, in accordance with the Institutional Animal Care Unit of the French National Institute of Health and Medical Research, the Ethics Committee of the University of Bordeaux (CE50, France), and the French Ministry of Higher Education, Research and Innovation (Authorization Nos. APAFIS#10936 and APAFIS#20053) in strict compliance with the French Ministry of Agriculture and Fisheries (Authorization No. B33-12-059) and the European Community Council Directive (2010/63/EU). Efforts were made to minimize animal suffering and reduce the number of mice used while maintaining reliable statistics.

### Locomotion Tests

Locomotion was measured in an open field (12,21) and with an Actimeter apparatus (Imetronic) (22) for THC and amphetamine, respectively.

### Viral Vector Procedures

To generate AAV-DIO-GSK-3β and AAV-DIO-GSK-3β^S9A^, the coding sequences for hemagglutinin (HA)–tagged GSK-3β and the respective S9A mutant were cloned into the pAAV-CAG-flex plasmid using standard molecular cloning techniques. The same pAAV-CAG-flex plasmid was used as an empty control (AAV-DIO-ctr). Mice were perfused transcardially with 4% paraformaldehyde, and their brains were processed for HA immunofluorescence coupled with in situ hybridization for the dopamine D_1_ receptor (D1R) or D_2_ receptor (D2R).

### Immunofluorescence and Double Fluorescence In Situ Hybridization Assay

The riboprobe D1-DIG antisense (1:1000) or D2-DIG antisense (1:1000) was used to detect mouse D1R or D2R messenger RNA (mRNA). The riboprobe D1-DIG or D2-DIG sense (1:1000) was used as a negative control (22).

### AlphaLISA Analyses

A detailed description of the AlphaLISA method was reported previously (23). Total cofilin, p-Akt1/2/3^T308^, p-Akt1/2/3^S473^, p-GSK-3β^S9^, p-mTOR^S2448^, p-RPS6^S240/244^, p-4EBP1^T37/46^, p-MEK1^S218/222^, p-ERK^1/2^ ^T202/Y204^ and total ERK^1/2^ levels were quantified using AlphaLISA SureFire Ultra kits according to PerkinElmer’s instructions.

### Cell Cultures

We used a neuronally derived cell line (STH*dh*^Q7/Q7^) from the mouse brain striatum. This cell line was derived from a knock-in transgenic mouse containing homozygous Huntingtin loci with a humanized Exon1 containing 7 polyglutamine (Gln in 3-letter code and Q in 1-letter code) repeats. The cells were transfected with *ARRB1* gene or plasmid complementary DNA3.1(+) and were treated with THC or its vehicle.

### Statistical Analyses

Statistical analyses were performed using GraphPad Prism software (version 7.05; GraphPad Software). See the Supplement for detailed statistical analyses.

## Results

### THC-Induced Hyperlocomotion Relies on CB1-Mediated Akt-GSK-3β Signaling Activity in the Dorsal Striatum of Mice

We have shown that the biased inhibition of CB1R by PREG or AEF0117 prevents THC-induced hyperactivity (12,21). Since these drugs do not alter CB1R G protein–mediated signaling, we focused on pathways independent of G proteins (17,24, 25, 26, 27) (Figure 1A) in the dorsal striatum, a brain structure critical for motor control and psychotic-related disorders (28,29).

**Figure 1:**
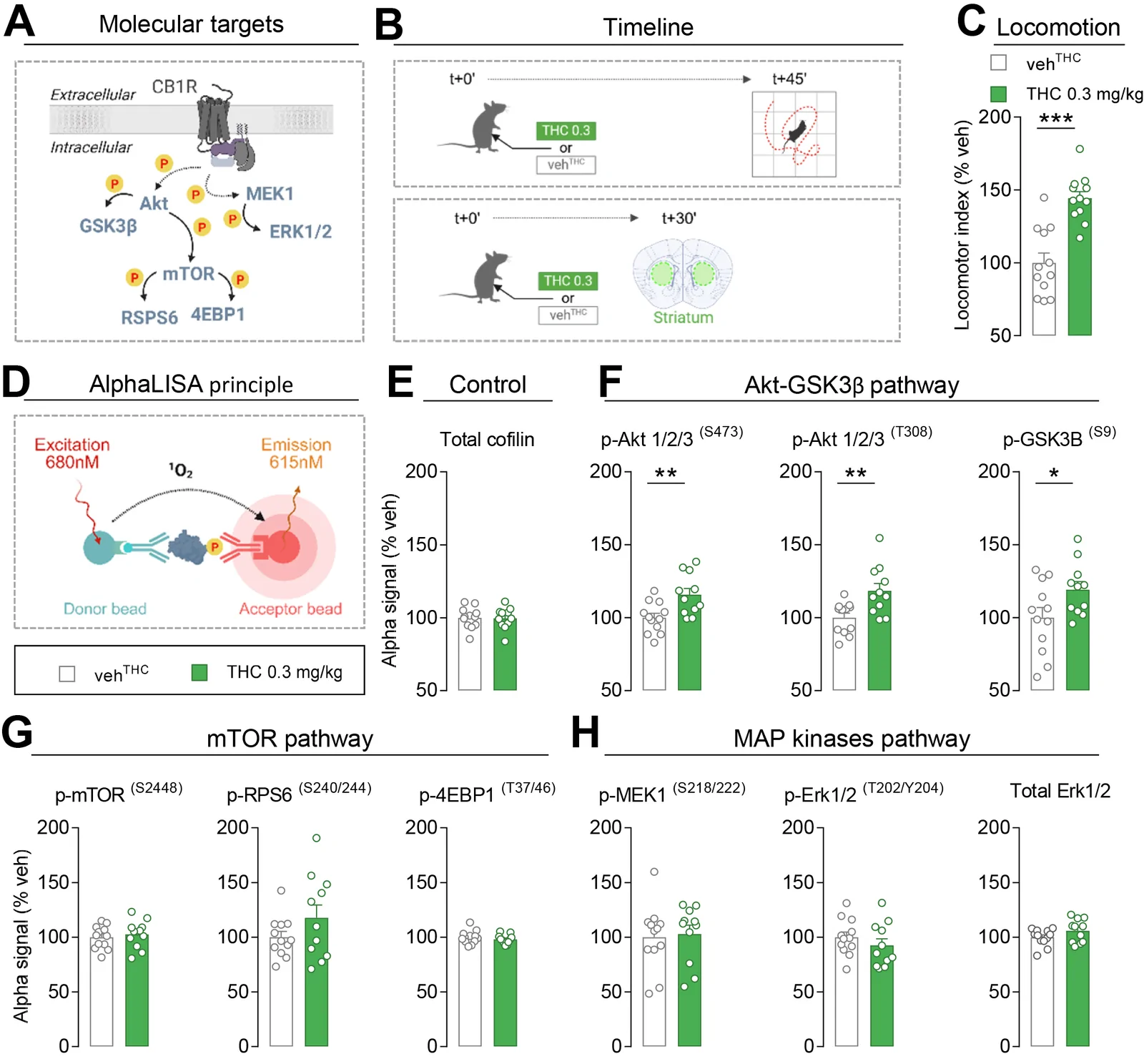
THC-induced hyperlocomotion is specifically associated with CB1R-mediated Akt-GSK-3β signaling activity in the dorsal striatum of mice. **(A)** Scheme of intracellular signaling pathways mediated by CB1R through sequential phosphorylation of kinases, including Akt and GSK-3β and Akt-downstream kinases mTOR, RPS6, and 4EBP1, as well as MEK1 and ERK^1/2^ kinases. **(B)** Timeline of locomotor activity assayed in an open-field (top) and dorsal striatum sampling (bottom) 45 and 30 minutes after intraperitoneal injection of THC (0.3 mg/kg) or veh^THC^, respectively. The behavioral assay and striatal sampling were performed on 2 separate groups of mice. **(C)** Effect of THC on locomotion in C57BL/6N mice (*n* = 12 per group) (*t*_22_ = 5.46, ∗∗∗*p* < .001). A locomotor index including horizontal and vertical locomotion (crosses + rearings) expressed as a percentage of the veh^THC^ was calculated. **(D)** Scheme of the principle of AlphaLISA technology for the quantitative detection of phosphorylated proteins. A detailed description of the principle has been reported previously (see the Supplement). **(E–H)** AlphaLISA assays of total and phosphorylated proteins from dorsal striatum protein extracts of C57BL/6N mice treated with veh^THC^ or THC (*n* = 11–12 per group). All assays were performed on the same tissue sample. Alpha signal is expressed as a percentage of veh^THC^. **(E)** Effect of THC on total cofilin, used as a loading control (*t*_21_ = 0.14, *p* = .089). **(F)** Effect of THC on phosphorylated Ser/Thr kinase Akt at 2 phosphorylation sites, the amino residues Ser 473 (Akt^S473^) (*t*_21_ = 3.04, ∗∗*p* < .01) and Thr 308 (Akt^T308^) (*t*_21_ = 3.13, ∗∗*p* < .01) and of GSK-3β at Ser 9 residue (GSK-3β^S9^) (*t*_21_ = 2.14, ∗*p* < .05). **(G)** Effect of THC on phosphorylated mTOR at Ser 2448 (*t*_21_ = 0.53, *p* = .6), RPS6 at Ser 240/244 (*t*_21_ = 1.44, *p* = .16) and of 4EBP1 at Thr 37/46 (*t*_21_ = 0.88, *p* = .39). **(H)** Effect of THC on phosphorylated MEK1 at Ser 218/222 (*t*_21_ = 0.86, *p* = .39) and ERK^1/2^ at Thr 202 and Tyr 204 (*t*_21_ = 0.97, *p* = .34), and the total ERK^1/2^ protein (*t*_21_ = 1.54, *p* = .13). Data are presented as mean ± SEM of single values. All statistical values are from a two-tailed, unpaired *t* test from THC vs. veh^THC^. 4EBP1, eukaryotic translation factor 4E-binding protein 1; CB1R, CB_1_ receptor; ERK^1/2^, extracellular signal-regulated protein kinase 1/2; GSK-3β, glycogen synthase kinase 3 beta; MEK1, mitogen-activated protein kinase/ERK 1; mTOR, mechanistic target of rapamycin; RPS6, 40S ribosomal protein S6; THC, Δ^9^-tetrahydrocannabinol; veh, vehicle.

Mice injected with a low dose of THC (Figure 1B) exhibited hyperlocomotion compared with vehicle-treated mice (*p* < .001) (Figure 1C). This hyperactivity was consistent with higher THC levels in striatal tissue (from 30 to 120 minutes post-THC; *p* < .001) (Figure S1). In a separate cohort of mice (Figure 1B), we mapped the intracellular pathways mediating THC-induced hyperlocomotion using AlphaLISA technology. This sensitive, high-throughput assay enables the simultaneous quantitative measurement of multiple protein phosphorylation states from small brain tissue samples (23) (Figure 1D). The levels of the housekeeping protein cofilin were not altered by THC injection (*p* = .089) (Figure 1E). THC increased the phosphorylation of Akt at the 2 sites, Ser 473 and Thr 308 (*p* < .01) and Akt downstream kinase GSK-3β at Ser 9 (*p* < .05) (Figure 1F), resulting in GSK-3β inhibition (30). In contrast, there was no change in the mTOR (mechanistic target of rapamycin) cascade, which can also be initiated by Akt (Figure 1A). THC did not alter the phosphorylation of mTOR (*p* = .6) or its downstream effectors, RPS6 (40S ribosomal protein S6) (*p* = .16) and 4EBP1 (eukaryotic translation factor 4E-binding protein 1) (*p* = .39) (Figure 1G). Finally, THC did not modify the phosphorylation of ERK^1/2^/MAPK kinase (*p* = .13), a prototypical marker of MAPK pathway activation in mammalian cells, or the upstream MEK1 (MAPK/ERK-1) protein (*p* = .39) (Figure 1A, H), which activates ERK^1/2^/MAPK (31). THC acts on the Akt-GSK-3β pathway without affecting other CB1R-mediated cascades, suggesting that THC-induced hyperlocomotion is mediated by Akt activation and subsequent GSK3β inhibition in the mouse dorsal striatum.

Then, we investigated whether the altered Akt-GSK-3β pathway plays a causal role in THC-induced hyperlocomotion using GDC-0084, a brain-penetrating drug that inhibits the pathway’s upstream target, PI3K (phosphoinositide 3-kinase) (32). First, we determined that 6 mg/kg was the highest subeffective dose of GDC-0084 that did not affect locomotion (*p* = .65) (Figure S2). Pretreatment with GDC-0084 was assessed for THC’s effects on locomotion and molecular signaling in mouse brain samples (Figure 2A). GDC-0084 prevented both THC-induced hyperlocomotion (*p* < .02) (Figure 2B) and THC-induced increases in phosphorylation/activation of Akt^S473/T308^ (*p* < .001 for both sites) and phosphorylation/inhibition of GSK-3β^S9^ (*p* < .01) in the dorsal striatum (Figure 2C). While GDC-0084 decreased basal Akt phosphorylation (*p* < .001), as previously reported (33), it had no effect per se on locomotion (*p* = .99) (Figure 2B) or GSK-3β^S9^ phosphorylation (*p* = .99) (Figure 2C). Extending our analysis to an anatomically and functionally interconnected motor circuit (34), GDC-0084 similarly prevented THC-induced increases in Akt^S473/T308^ activation and GSK-3β^S9^ inhibition in the ventral striatum (nucleus accumbens) (Akt^S473/T308^: *p* < .001; GSK-3β^S9^: *p* < .01) and cerebellum (Akt^S473/T308^: *p* < .001; GSK-3β^S9^: *p* < .001) (Figure S3). Together, these findings indicate that CB1R-dependent Akt/GSK-3β signaling across the cerebello–basal ganglia network is required for a low dose of THC-induced hyperlocomotion.

**Figure 2:**
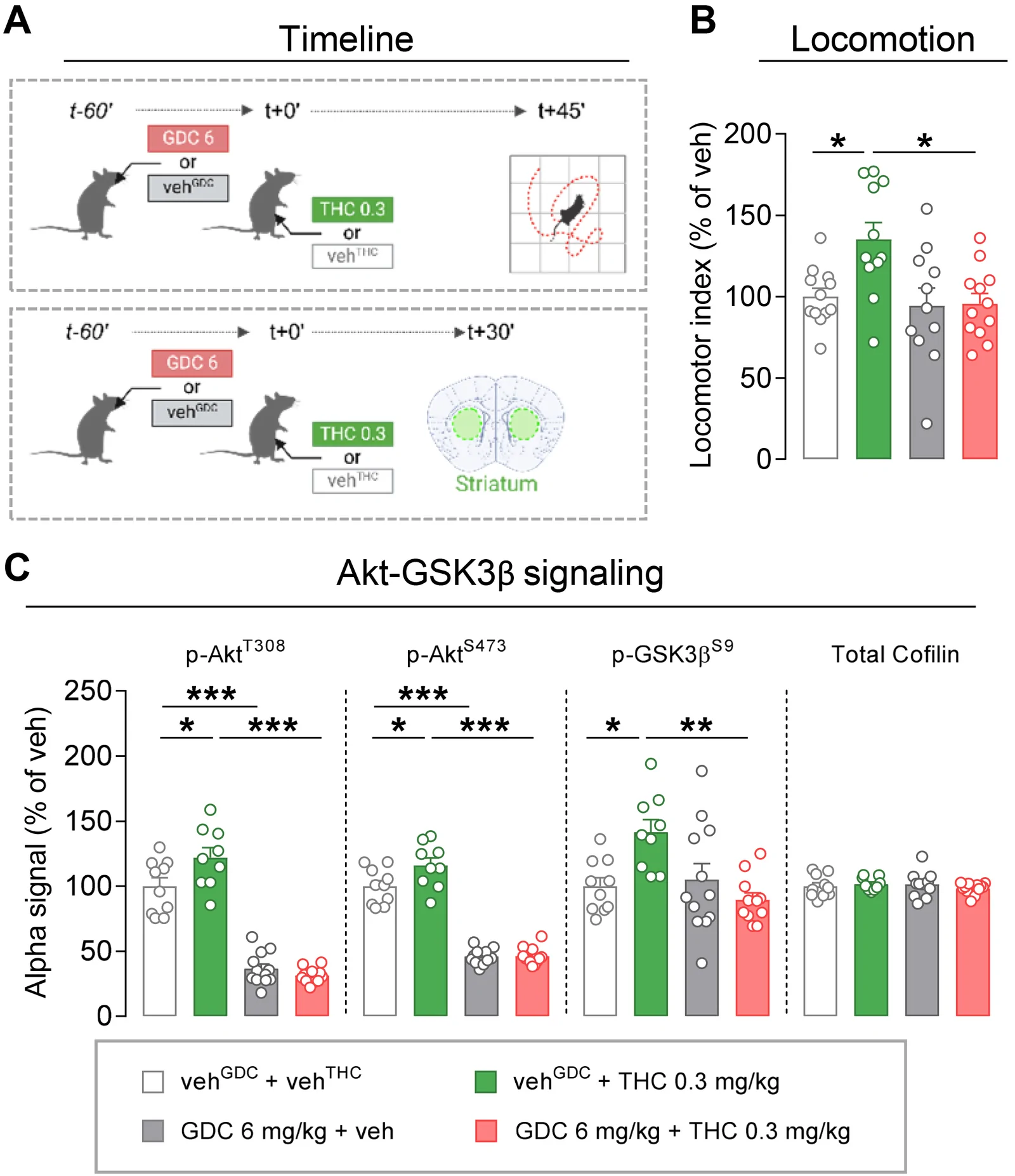
GDC-0084 blocks the effects of THC on locomotion and Akt-GSK-3β pathway activity in mice. **(A)** Timeline of locomotor activity assayed in an open-field (top) and dorsal striatum sampling (bottom) 45 and 30 minutes after intraperitoneal injection of THC (0.3 mg/kg) or veh^THC^, respectively with a 1-hour pretreatment of oral administration of GDC-0084 (6 mg/kg) or veh^GDC^. The behavioral assay and striatal sampling were performed on 2 separate groups of mice. **(B)** Effect of GDC-0084 (6 mg/kg) on locomotion in C57BL/6N mice treated with THC or veh^THC^ (*n* = 12 per group) (*F*_1,42_ = 4.1, *p* < .05; veh^veh^ vs. veh^THC^: ∗*p* < .02; veh^veh^ vs. veh^GDC^: *p* = .99; veh^THC^ vs. GDC^THC^: ∗*p* < .02). A locomotor index including horizontal and vertical locomotion (crosses + rearings) expressed as a percentage of the veh^THC^ was calculated. **(C)** Effect of GDC-0084 (6 mg/kg) on phosphorylation of Akt at Thr 308 and Ser 473 and of GSK-3β at Ser 9, and total cofilin in dorsal striatum extracts from C57BL/6N mice treated with THC (0.3 mg/kg) or veh^THC^ (*n* = 9–12 per group) (p-Akt^T308^: *F*_1,39_ = 6, *p* < .02; veh^veh^ vs. veh^THC^: ∗*p* < .05, veh^veh^ vs. veh^GDC^: ∗∗∗*p* < .001, veh^THC^ vs. GDC^THC^: ∗∗∗*p* < .001; p-Akt^S473^: *F*_1,39_ = 5.4, *p* < .05; veh^veh^ vs. veh^THC^: ∗*p* < .02, veh^veh^ vs. veh^GDC^: ∗∗∗*p* < .001, veh^THC^ vs. GDC^THC^: ∗∗∗*p* < .001, p-GSK-3β^S9^: *F*_1,39_ = 6.6, *p* < .02; veh^veh^ vs. veh^THC^: ∗*p* < .05, veh^veh^ vs. veh^GDC^: *p* = .99; veh^THC^ vs. GDC^THC^: ∗∗*p* < .01; total cofilin: *F*_1,39_ = 1.1, *p* = .30). All assays were performed on the same tissue sample. Alpha signal is expressed as a percentage of veh^THC^. Data are presented as mean ± SEM of single values. All statistical values are from a 2-way analysis of variance, followed by Sidak’s multiple comparison test. THC, Δ^9^-tetrahydrocannabinol; veh, vehicle.

### β-Arrestin 1 Is Involved in THC-Induced Akt-GSK-3β Signaling Activity in Striatal Cell Lines

We explored the CB1R-mediated intracellular cascade further, analyzing which upstream signal transducer may sustain GSK-3β signaling. Beyond classical GPCR desensitization and internalization, β-arrestin proteins have emerged as signal transducers. While β-arrestin 2 is involved in the desensitization and internalization of receptors, β-arrestin 1 specifically initiates signaling cascades rather than driving internalization (35). Therefore, we investigated whether the β-arrestin 1 protein mediates the alteration of Akt-GSK-3β by THC. Experiments were performed using the STH*dh*^Q7/Q7^ murine striatal medium spiny neuron (MSN) cell line, an appropriate model for studying CB1R-biased signaling as it endogenously expresses CB1R and exhibits biased signaling (36,37), including β-arrestin 1–mediated Akt^S473^ phosphorylation (38). Using the highly accurate droplet digital polymerase chain reaction (39), we found low basal expression of the β-arrestin 1–encoding gene (*Arrb1*) in our STH*dh*^Q7/Q7^ clone relative to other signal transducers (Figure S4A), contrasting with previous reports (38). To overcome this, we transfected STH*dh*^Q7/Q7^ cells with a plasmid expressing the human β-arrestin 1 protein (Figure 3A), which increased ARRB1 mRNA expression (*p* < .001) (Figure S4B). Upon ARRB1 expression, THC significantly increased the phosphorylation of Akt^T308^ (*p* < .01), Akt^S473^ (*p* < .05), and GSK-3β^S9^ (*p* < .02), but not in empty vector control STH*dh*^Q7/Q7^ cells (Akt^T308^: *p* = .90, Akt^S473^: *p* = .65, and GSK-3β^S9^: *p* = .96) (Figure 3B). These findings suggest that β-arrestin 1 is required for THC-driven Akt-GSK-3β activation in mouse striatal cells.

**Figure 3:**
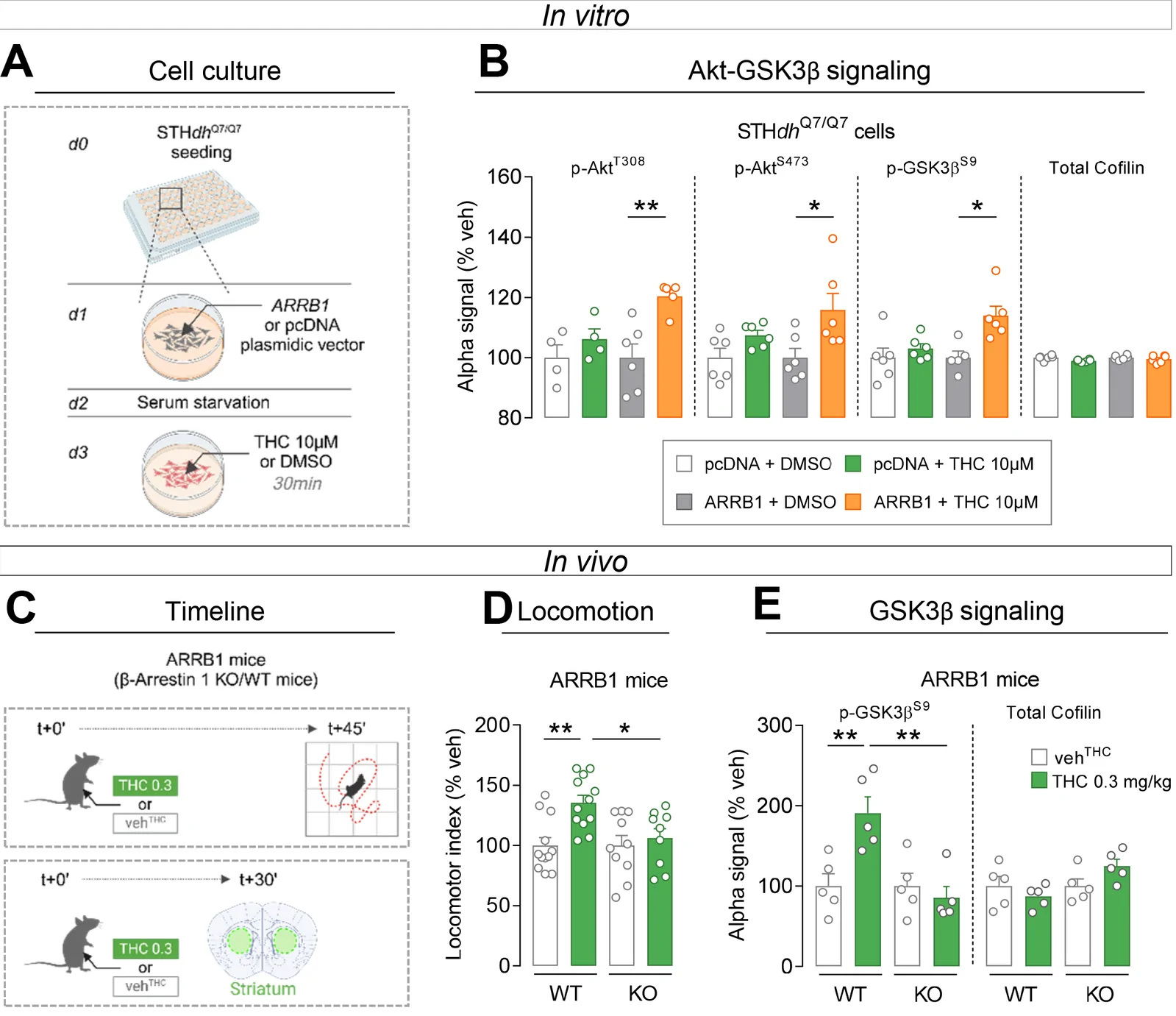
β-arrestin 1 is required for THC effects on Akt-GSK-3β signaling activity in cell culture, on locomotor activity in mice, and on GSK-3β phosphorylation in mouse striatal tissue. **(A)** STH*dh*^Q7/Q7^ cell transfection timeline. On day 0, STH*dh*^Q7/Q7^ cells were seeded in 96-well plates. After 24-hours, STH*dh*^Q7/Q7^ cells were transfected with *ARRB1* gene encoding human β-arrestin 1 or empty pcDNA vectors. After 24 hours of serum starvation, cells were treated with THC (10 μM) or DMSO (vehicle) for 30 minutes before the AlphaLISA assay. **(B)** Effect of THC on the phosphorylation of Akt at Thr 308 and Ser 473, GSK-3β at Ser 9, and total cofilin in STH*dh*^Q7/Q7^ cells transfected with pcDNA or *ARRB1* gene (*n* = 4–6 per group). (p-Akt^T308^: *F*_1,15_ = 11.2, *p* < .01; pcDNA^veh^ vs. pcDNA^THC^: *p* = .90, *ARRB1*^veh^ vs. *ARRB1*^THC^: ∗∗*p* < .01; p-Akt^S473^: *F*_1,20_ = 10.3, *p* < .01; pcDNA^veh^ vs. pcDNA^THC^: *p* = .65, *ARRB1*^veh^ vs. *ARRB1*^THC^: ∗*p* < .05; p-GSK-3β^S9^: *F*_1,19_ = 9.8, *p* < .01; pcDNA^veh^ vs. pcDNA^THC^: *p* = .96, *ARRB1*^veh^ vs. *ARRB1*^THC^: ∗*p* < .02; total cofilin: *F*_1,20_ = 4.6, *p* < .05; pcDNA^veh^ vs. pcDNA^THC^: *p* = .26, *ARRB1*^veh^ vs. *ARRB1*^THC^: *p* = .92). All assays were performed on the same tissue sample. The Alpha signal is expressed as a percentage of veh^THC^. **(C)** Timeline of locomotor activity assayed in an open-field (top) and dorsal striatum sampling (bottom) 45 and 30 minutes after intraperitoneal injection of THC (0.3 mg/kg) or veh^THC^ in β-arrestin 1 KO (*Arrb1*^−/−^) mice and WT littermates. The behavioral assay and striatal sampling were performed on 2 separate groups of mice. **(D)** Effect of THC on locomotor activity in WT and KO mice (*n* = 10–12 per group) (*F*_1,39_ = 4.2, *p* < .05; WT^veh^ vs. WT^THC^: ∗∗*p* < .01, KO^veh^ vs. KO^THC^: *p* = .99, WT^THC^ vs. KO^THC^: ∗*p* < .05). A locomotor index including horizontal and vertical locomotion (crosses + rearings) expressed as a percentage of veh^THC^ was calculated. **(E)** Effect of THC on the phosphorylation of GSK-3β at Ser 9 and total cofilin in dorsal striatum extracts from WT and KO mice (*n* = 5–6 per group) (p-GSK-3β^S9^: *F*_1,16_ = 10.1, *p* < .05; WT^veh^ vs. WT ^THC^: ∗∗*p* < .01, KO^veh^ vs. KO^THC^: *p* = .99, WT ^THC^ vs. KO^THC^: ∗∗*p* < .01; total cofilin: *F*_1,16_ = 4.3, *p* = .054). The alpha signal is expressed as a percentage of veh^THC^. Data are presented as mean ± SEM of single values. All statistical values are from a 2-way analysis of variance followed by Sidak’s multiple comparison test. GSK-3β, glycogen synthase kinase 3 beta; KO, knockout; pcDNA, plasmid complementary DNA; THC, Δ^9^-tetrahydrocannabinol; veh, vehicle; WT, wild-type.

### β-Arrestin 1 Mediates THC-Induced Hyperlocomotion and Increased GSK-3β Phosphorylation in Mice

To confirm that β-arrestin 1 mediates THC-induced hyperlocomotion through the Akt-GSK-3β pathway, locomotion was assessed in β-arrestin 1–deficient (*Arrb1*^−/−^) mice (40) (Figure 3C). Strikingly, THC significantly increased locomotor activity in wild-type (WT) mice (*p* < .01), but had no effect in *Arrb1*^−/−^ littermates (*p* = .92) (Figure 3D). This behavioral deficit was specific to THC, as amphetamine-induced hyperlocomotion occurred in both genotypes (*p* < .001) (Figure S5), thereby confirming that β-arrestin 1 is necessary to mediate THC-induced hyperlocomotion. At the molecular level, THC increased p-GSK-3β^S9^ in WT mice, but not in *Arrb1*^−/−^ littermates, across the dorsal striatum (WT mice: *p* < .01; *Arrb1*^−/−^ mice: *p* = .99) (Figure 3E), nucleus accumbens (WT mice: *p* < .05; *Arrb1*^−/−^ mice: *p* = .97), and cerebellum (WT mice: *p* < .05; *Arrb1*^−/−^ mice: *p* = .99) (Figure S6). These findings demonstrate that β-arrestin 1 is causally required for THC-induced hyperlocomotion through CB1R Akt-GSK-3β signaling.

### THC-Induced Hyperlocomotion Involves CB1R Located on Striatal D2 Neurons

Next, we identified the localization of CB1R and the specific neuronal populations expressing CB1R in the dorsal striatum that drive low-dose THC-induced hyperlocomotion. Besides being localized at the plasma membrane, CB1Rs are intracellularly associated with different organelles, including mitochondria CB1R (mtCB1R), where they control several cellular functions (41,42). Because striatal mtCB1R mediates cannabinoid-induced hypolocomotion (43,44), we first tested the newly generated and characterized DN22-CB1-KI mice, which lack mtCB1Rs (43,44). THC induced normal hyperlocomotion in both WT (*p* < .05) and DN22-CB1-KI (*p* < .01) mice (Figure 4A), ruling out mtCB1R involvement in increased locomotion. Then, we investigated the cellular location of the CB1R involved in THC-induced hyperlocomotion, focusing on its high expression in the terminals of striatal MSNs (22,44,45) projecting to the globus pallidus or substantia nigra reticulata (SNr) (46, 47, 48). THC did not induce hyperlocomotion in conditional mutant mice lacking CB1R in GABAergic (gamma-aminobutyric acidergic) neurons, including MSNs (13,45) (*p* = .50), unlike their WT littermates (*p* < .02) (Figure 4B), suggesting that CB1Rs at GABA neurons, including MSNs, are necessary for the enhanced locomotion by THC. Then, we examined whether this effect depends on one of the 2 subtypes of MSNs, the direct (striatonigral) and indirect (striatopallidal) pathways, which regulate movement and action (49, 50, 51, 52) and in which CB1R subpopulations may be involved in motor behavior (52). The direct pathway originates from D1-expressing MSNs (D1-MSNs) projecting primarily to the SNr and globus pallidus internae (GPi). The indirect pathway originates from D2-expressing MSNs (D2-MSNs) and adenosine A2a receptors, projecting to the globus pallidus externae (GPe), which can target the subthalamic nucleus and GPi/SNr (49,53). We used the validated conditional mouse lines Drd1-CB1-KO and Adora2a-CB1-KO, which lack CB1R in D1-MSNs or in D2-MSNs, respectively (22). THC-induced hyperlocomotion was observed in WT mice of both genotypes (*p* < .001), as well as in Drd1-CB1-KO mice (*p* < .01) (Figure 4C), but not in Adora2a-CB1-KO mice (*p* > .99) (Figure 4D). These results strongly indicate that CB1Rs at D2-MSNs, rather than D1-MSNs, are necessary for low-dose THC-induced hyperlocomotion. The lack of hyperactivity in Adora2a-CB1-KO mice is not due to a motor deficit, as these mice exhibit normal motor activity and amphetamine-induced hyperlocomotion (22). Our data suggest that CB1R at D2-MSNs may underlie THC-induced hyperlocomotion, as CB1 protein levels are reduced in the GPe of Adora2a-CB1-KO mice compared with WT littermates (22), and potentiation of D2-MSN activity by the D2R antagonist haloperidol (29) counteracts the increase in locomotor activity induced by THC (*p* < .01) (Figure S7). Finally, we aimed to determine whether GSK-3β phosphorylation at S9 by Akt in D2-MSNs is required for THC-induced hyperlocomotion. We injected a viral vector (i.e., AAV-DIO-GSK-3β^S9A^) expressing a Cre-dependent HA-tagged dominant negative mutant form of GSK-3β bearing a point mutation that converts Ser 9 to Alanine (i.e., GSK-3β^S9A^), thus preventing its phosphorylation, in the dorsal striatum of Tg(Adora2a∗cre) KG139Gsat mouse line (Figure 4E). First, we confirmed, by HA immunodetection coupled with fluorescent in situ hybridization against D1R or D2R, that the dominant negative GSK-3β^S9A^ protein was not expressed in D1-expressing neurons but rather was selectively expressed in D2-expressing neurons (Figure 4F). Virally disabling GSK-3β phosphorylation (i.e., GSK-3β^S9A^) in D2-MSNs prevented THC-induced hyperlocomotion (GSK-WT mice: *p* < .01; GSK-S9 mice: *p* > .99) (Figure 4G). Overall, our findings indicate that GSK-3β inactivation is a sufficient condition to mediate THC-induced hyperlocomotion through CB1R at D2-MSNs.

**Figure 4:**
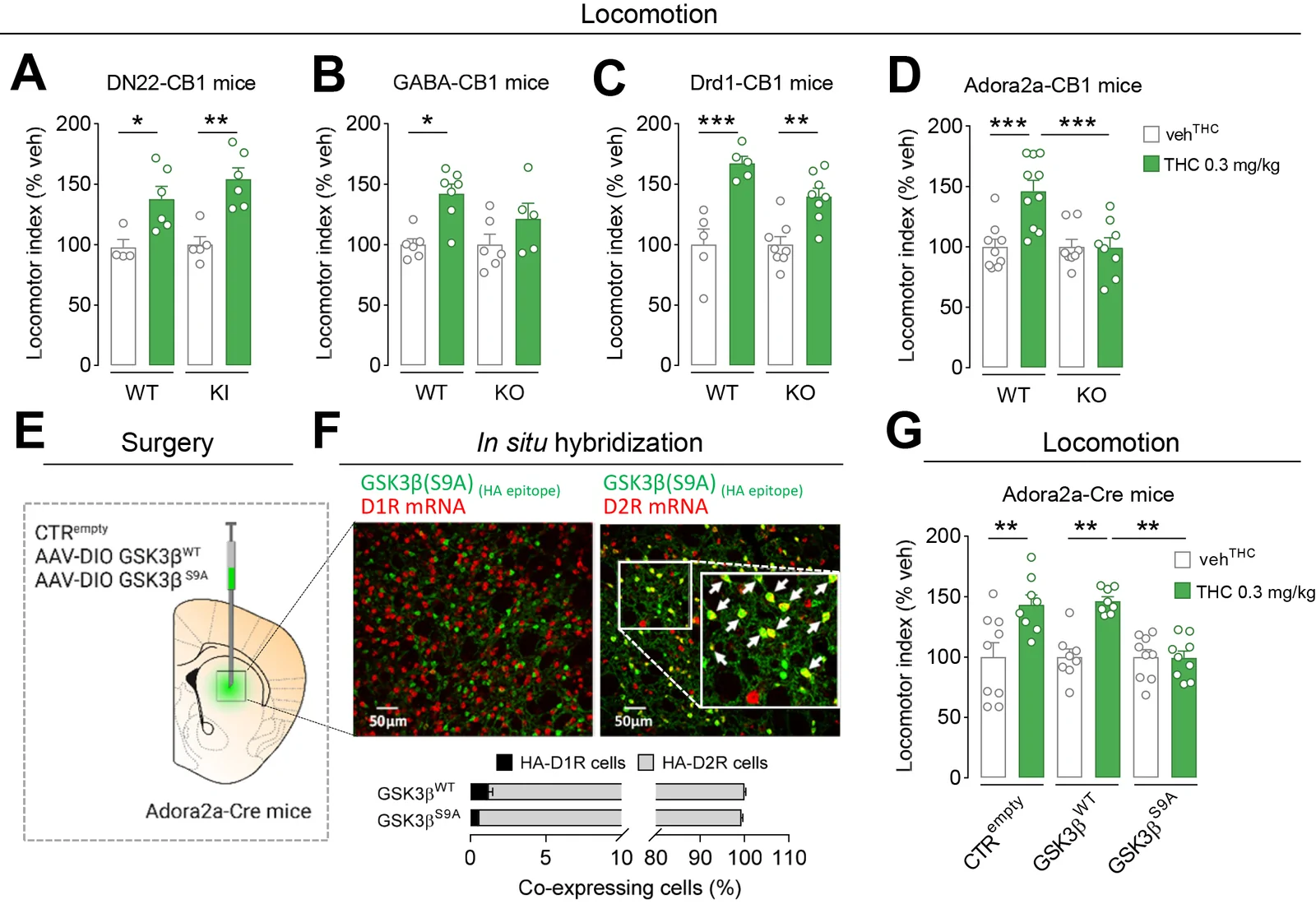
CB1R located on plasma membrane of striatal D2 neurons trigger THC-induced hyperlocomotion in mice. The locomotor index, including horizontal and vertical locomotion (crosses + rearings), is expressed as a percentage of the veh^THC^. **(A)** Locomotion in DN22-CB1-KI mice, lacking mitochondrial CB1R (*n* = 4–6 per group) (*F*_1,17_ = 27, *p* < .001; WT^veh^ vs. WT^THC^: ∗*p* < .05, KI^veh^ vs. KI^THC^: ∗∗*p* < .01, WT^VEH^ vs. KI^VEH^: *p* > .99, WT^THC^ vs. KI^THC^: *p* = .71). **(B)** Locomotion in GABA-CB1-KO mice, lacking CB1R on GABAergic neurons (*n* = 5–7 per group) (*F*_1,20_ = 13, *p* < .01; WT^veh^ vs. WT^THC^: ∗*p* < .02, KO^veh^ vs. KO^THC^: *p* = .50, WT^VEH^ vs. KO^VEH^: *p* > .99, and WT^THC^ vs. KO^THC^: *p* = .49). **(C)** Locomotion in Drd1-CB1-KO mice, lacking the CB1R at the terminals of D1-MSNs (in the substantia nigra) (*n* = 5–8 per group) (*F*_1,22_ = 42, *p* < .001; WT^veh^ vs. WT^THC^: ∗∗∗*p* < .001, KO^veh^ vs. KO^THC^: ∗∗*p* < .01, WT^VEH^ vs. KO^VEH^: *p* > .99, WT^THC^ vs. KO^THC^: *p* = .15). **(D)** Locomotion in Adora2a-CB1-KO mice, lacking the CB1R at the terminals of D2-MSNs (in the globus pallidus externae) (*n* = 8–10 per group) (*F*_1,31_ = 9.2, *p* < .001; WT^veh^ vs. WT^THC^: ∗∗∗*p* < .001; KO^veh^ vs. KO^THC^: *p* > .99; WT^VEH^ vs. KO^VEH^: *p* > .99; WT^THC^ vs. KO^THC^: ∗∗∗*p* < .001). **(E)** Surgical scheme for viral injection of control virus (CTR^empty^), AAV-DIO-GSK-3β^WT^ or AAV-DIO-GSK-3β^S9A^ in the dorsal striatum of Adora2a-Cre mice. **(F)** Illustrations of immunofluorescence and double fluorescent in situ hybridization experiments (top). GSK-3β^S9A^ (green) and D1R mRNA (left) and D2R mRNA (right) (red). Scale bars, 50 μm. Relative quantification of coexpressing cells (yellow) of GSK-3β^WT^ and GSK-3β^S9A^ for HA-D1R and HA-D2R cells (bottom). **(G)** Effect of THC on locomotor activity in Adora2a-Cre mice injected with CTR^empty^, GSK-3β^WT^ or GSK-3β^S9A^ viruses into the dorsal striatum (*n* = 8–9 per group) (*F*_2,45_ = 6.2, *p* < .01; CTR^veh^ vs. CTR^THC^: ∗∗*p* < .01, GSK-WT^veh^ vs. GSK-WT^THC^: ∗∗*p* < .01, GSK-S9A^veh^ vs. GSK-S9A^THC^: *p* > .99, GSK-WT^THC^ vs. GSK-S9A^THC^: ∗∗*p* < .01). The locomotor index, including horizontal and vertical locomotion (crosses + rearings), is expressed as a percentage of the veh^THC^. Data are presented as mean ± SEM of single values. All statistical values are from a 2-way analysis of variance followed by Sidak’s multiple comparison test. CB1R, CB_1_ receptor; CTR, control; GABA, gamma-aminobutyric acid; KI, knock-in; KO, knockout; mRNA, messenger RNA; MSN, medium spiny neuron; THC, Δ^9^-tetrahydrocannabinol; veh, vehicle; WT, wild-type.

### PREG and AEF0117 Prevent THC-Induced GSK-3β–Dependent Hyperlocomotion in Mice

We evaluated whether the CB1-SSi compounds, PREG and AEF0117, prevent THC-induced hyperlocomotion by targeting the CB1-dependent GSK-3β pathway (Figure 5A, D). Confirming previous findings (12), PREG blocked THC-induced hyperlocomotion (*p* < .01) without affecting baseline locomotion (*p* > .99) (Figure 5B). At the molecular level, PREG prevented the THC-induced increase in GSK-3β^S9^ phosphorylation across the dorsal striatum (*p* < .02), nucleus accumbens (*p* < .001), and cerebellum (*p* < .01), without having any effect per se in all brain areas (*p* = .78, *p* = .99, and *p* = .99, respectively) (Figure 5C and Figure S8A, B). Although our PREG injection does not increase other steroid levels in rodents (11), PREG is a natural steroid precursor that can be metabolized into downstream steroids when orally administered to humans (54). To rule out this possibility, we tested the synthetic PREG derivative, AEF0117, which does not convert into PREG-downstream steroids, has a long half-life, and is orally bioavailable, making it a promising therapeutic drug (21). Confirming previous findings (21), AEF0117 blocked THC-induced hyperlocomotion in mice (*p* < .001) without altering locomotion per se (*p* = .98) (Figure 5E). Furthermore, AEF0117 prevented THC-induced GSK-3β^S9^ phosphorylation across the dorsal striatum (*p* < .001), nucleus accumbens (*p* < .01), and cerebellum (*p* < .01) without affecting it per se in any brain area (*p* = .46, *p* = .99, and *p* = .99, respectively) (Figure 5F and Figure S8C, D). These data indicate that PREG and AEF0117 prevent the increase in locomotor activity induced by a low dose of THC by targeting the CB1R GSK-3β signaling pathway, revealing a novel mechanism of action for AEF0117.

**Figure 5:**
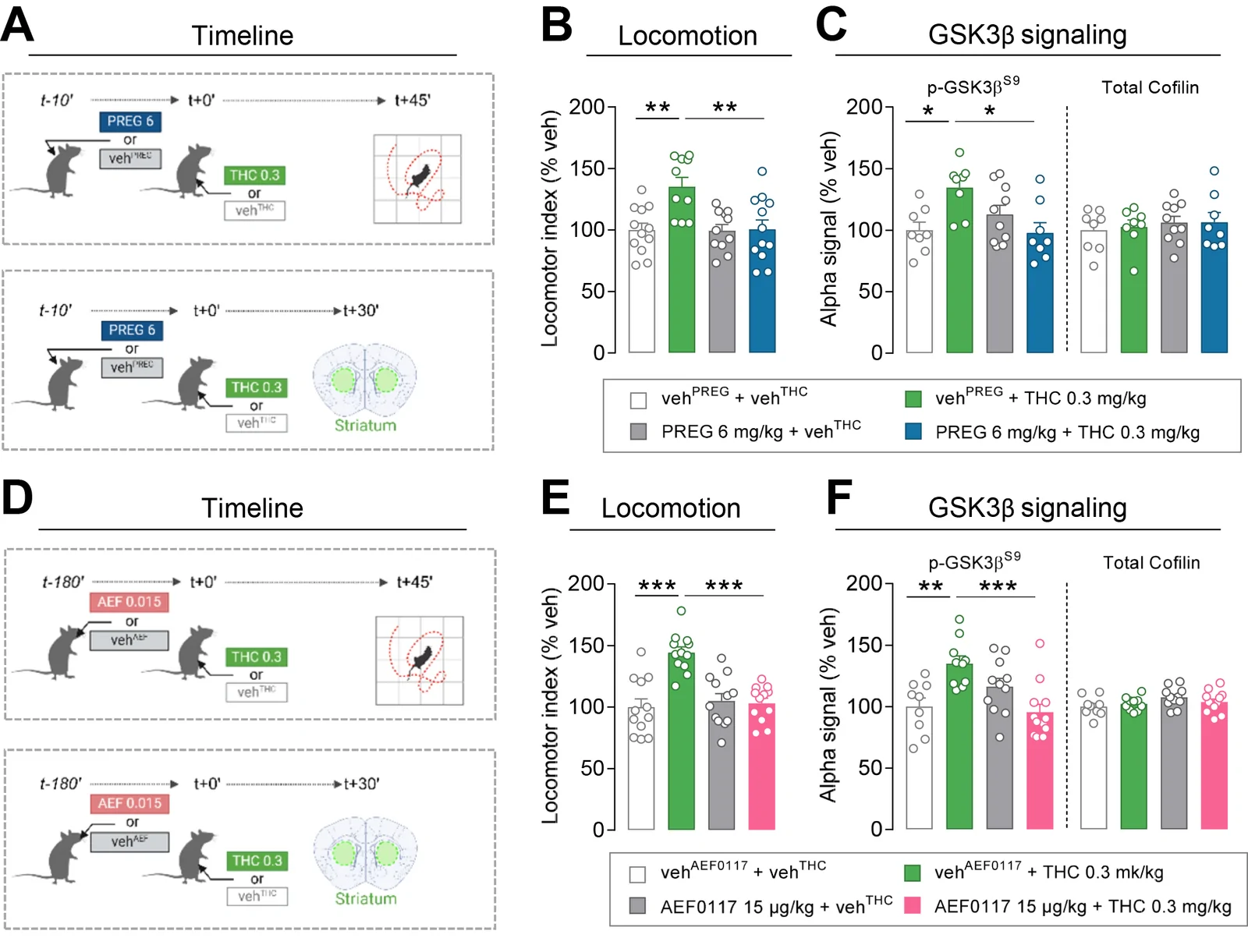
PREG and AEF0117 reverse CB1R-mediated effects of THC on locomotion and Akt-GSK-3β pathway activity. **(A, D)** Timeline of locomotion assayed in an open-field (top) and dorsal striatum sampling (bottom) 45 and 30 minutes after intraperitoneal injection of THC (0.3 mg/kg) or veh^THC^, respectively, with **(A)** a 10-minute pretreatment with subcutaneous injection of PREG (6 mg/kg) or veh^PREG^ or **(D)** a 3-hour pretreatment with oral administration of AEF0117 (0.015 mg/kg or 15 μg/kg) or veh^AEF0117^. The pretreatment effects of PREG and AEF0117 were assessed in separate groups of mice. Behavioral assays and striatal sampling were also performed on separate groups of mice. **(B, E)** The locomotor index, including horizontal and vertical locomotion (crosses + rearings), is expressed as a percentage of the veh^THC^. **(B)** Effect of PREG on locomotion in C57BL/6N mice treated with THC or veh^THC^ (*n* = 10–12 per group) (*F*_1,41_ = 6.8, *p* < .02; veh^veh^ vs. veh^THC^: ∗∗*p* < .01, veh^veh^ vs. PREG^VEH^: *p* > .99, veh^THC^ vs. PREG^THC^: ∗∗*p* < .01, PREG^VEH^ vs. PREG^THC^: *p* > .99). **(E)** Effect of AEF0117 on locomotion in C57BL/6N mice treated with THC or veh^THC^ (*n* = 12 per group) (*F*_1,44_ = 18, *p* < .001; veh^veh^ vs. veh^THC^: ∗∗∗*p* < .001, veh^veh^ vs. AEF0117^VEH^: *p* = .98, veh^THC^ vs. AEF0117^THC^: ∗∗∗*p* < .001, AEF0117^VEH^ vs. AEF0117^THC^: *p* > .99). **(C, F)** GSK-3β signaling was assessed by phosphorylation of GSK-3β at Ser 9 (p-GSK-3β^S9^). **(C)** Effect of PREG on p-GSK-3β^S9^ in dorsal striatum extracts from C57BL/6N mice treated with THC or veh^THC^ (*n* = 8–10 per group) (*F*_1,30_ = 11, *p* < .01; veh^veh^ vs. veh^THC^: ∗*p* < .02, veh^veh^ vs. PREG^VEH^: *p* = .78, veh^THC^ vs. PREG^THC^: ∗*p* < .02, PREG^VEH^ vs. PREG^THC^: *p* = .64). **(F)** Effect of AEF0117 on p-GSK-3β^S9^ in dorsal striatum extracts from C57BL/6N mice treated with THC or veh^THC^ (*n* = 9–12 per group) (*F*_1,38_ = 18, *p* < .001; veh^veh^ vs. veh^THC^: ∗∗*p* < .01, veh^veh^ vs. AEF0117^VEH^: *p* = .46, veh^THC^ vs. AEF0117^THC^: ∗∗∗*p* < .001, AEF0117^VEH^ vs. AEF0117^THC^: *p* = .14). For all AlphaLisa analyses, there was no difference in total cofilin between groups: *F*_1,30_ = 0.03, *p* = .86 **(C)** and *F*_1,38_ = 1.6, *p* = .21 **(D)**. p-GSK-3β^S9^ and total cofilin assays were performed on the same tissue sample. Data are expressed as a percentage of veh^THC^. All statistical values are from a 2-way analysis of variance, followed by a Sidak’s multiple comparison test. CB1R, CB_1_ receptor; GSK-3β, glycogen synthase kinase 3 beta; PREG, pregnenolone; THC, Δ^9^-tetrahydrocannabinol; veh, vehicle.

## Discussion

This study showed that low-dose THC triggered hyperlocomotion via a striatal CB1R–β-arrestin 1/Akt/GSK-3β pathway within D2-MSNs. Furthermore, we demonstrated that targeting this cascade mediated the inhibitory effect of the neurosteroid PREG and its clinical-grade analog, the first-in-class CB1-SSi, AEF0117.

### Relevance of the Acute THC Dose

Our preclinical study design, using an acute low dose of THC, modeled the psychostimulant and psychomimetic effects of acute cannabis exposure in healthy humans or in occasional cannabis users, which can persist beyond the period of acute intoxication (10,55, 56, 57). This aligns with the DSM-5-TR classification for cannabis-induced psychotic disorders (58), supported by meta-analyses showing that low doses of acute THC induce positive-like psychotic symptoms (10,59, 60, 61). This is relevant to reports that psychotogenic drugs cause hyperlocomotion in rodents, suggesting that this effect may serve as a marker of positive psychotic symptoms (5,12).

To correlate locomotion with THC levels, we quantified THC in plasma and the dorsal striatum, a key structure integrating information relevant to adaptive motor control and psychotic-like dysfunction (28,29). We used a validated, high-throughput liquid chromatography–tandem mass spectrometry assay (62), considered the method of choice for assessing brain-to-blood drug distribution (63). The peak plasma THC level (Figure S1A) mirrors levels that impair human neurocognitive performance, including perceptual motor control and motor inhibition (57). While plasma levels peaked at T30 minutes, which is consistent with slower plasma clearance following intraperitoneal administration than with other modes of administration (64,65), striatal THC peaked at T120 minutes (Figure S1B). This blood-brain distribution timeframe matches prior rodent pharmacokinetic studies (21,64,66) that used methods comparable to those in our study, including THC formulations and routes of administration, which are 2 key determinants of THC pharmacokinetics and tissue distribution (67,68). Crucially, despite the delayed peak, striatal THC was already significant at T30 minutes, validating this time point for analyzing striatal proximal CB1R signaling before the behavioral readout (T45 minutes). Assessing early signaling at T30 minutes captures the primary CB1R signaling activation underlying hyperlocomotion, while avoiding secondary confounding effects that could emerge later (e.g., T120 minutes). These late-stage confounds include feedback loops, signaling crosstalk, THC-induced hypolocomotion, and environment-driven changes in protein phosphorylation (e.g., from novelty exposure during locomotor testing) (16, 69).

### Posttranslational Modifications—Which Cell Signaling and Where

Posttranslational modifications, particularly protein phosphorylation, dynamically alter protein function to drive cellular and behavioral changes (70). Using high-throughput alpha assays (23), we identified a novel CB1R mechanism in which β-arrestin 1 recruits an Akt/GSK-3β signaling cascade in D2-MSNs to mediate low-dose THC-induced hyperlocomotion. This behavioral response was preceded by Akt^T308/S473^ phosphorylation/activation and GSK-3β^S9^ phosphorylation/inhibition (Figure 1). GSK-3β acts as a versatile signal hub for GPCR-mediated pathways, with its functional specificity governed by its subcellular localization, substrate availability, and interacting receptors (30). Notably, reducing its activity by increasing its phosphorylation state in striatopallidal neurons proved essential for THC-induced hyperlocomotion. Indeed, upstream PI3K inhibition with the brain-penetrant compound GDC-0084 (32,33) selectively blocked the effect of THC on locomotor activity and on striatal phosphorylated Akt/GSK-3β (Figure 2). Although GDC-0084 does not alter baseline locomotion, the observed reduction in basal Akt phosphorylation, consistent with previous findings in the brains of mice treated with a 25 mg/kg oral dose, likely reflects a general PI3K/mTOR pathway modulation. By targeting PI3K, an upstream kinase, GDC-0084 modulates both the Akt-GSK3β and mTOR signaling cascades. However, this pathway modulation cannot explain the rescue of THC-induced hyperactivity by GDC-0084 (which is mediated by striatal D2-MSNs), as striatal mTOR signaling remained unaffected following THC treatment (Figure 1G). Furthermore, the role of mTOR in motor coordination, running activity, and accelerated motor skill learning is predominantly associated with striatal D1-MSNs rather than D2-MSNs (71, 72, 73).

Our in vitro and in vivo findings establish that β-arrestin 1 acts as a mandatory signal transducer for CB1R-driven motor stimulation (Figure 3). Consistent with previous reports of CB1R functional selectivity, emphasizing G protein–independent pathways in CB1R-mediated behavioral responses, the loss of THC response in *Arrb1* knockout (KO) mice, which do not constitutively express β-arrestin 1, confirms that β-arrestin 1–biased signaling mediates CB1R-GSK-3β–dependent psychomotor stimulation.

The dose-dependent effects of THC on locomotor activity reflect distinct cellular and subcellular CB1R pools. While CB1Rs in D1-expressing cells (including D1-MSNs) mediate THC-induced catalepsy and motor deficits in mice (22,44,45,74), CB1Rs at striatopallidal D2-MSNs are involved in amphetamine-induced locomotion (22,75). This aligns with the distinct classical roles of striatal D1-MSNs and D2-MSNs in motor control, though both pathways may be functionally related (50,52). Accordingly, we hypothesized that THC-induced hyperlocomotion relies on CB1R in specific striatal neuronal populations. We show that CB1Rs on GABAergic D2-MSNs terminals, but not D1-MSNs, are specifically required for this effect. This mechanism parallels D2R-mediated motor control, where increased dopaminergic tone engages the striatal Akt-GSK-3β pathway (76). Functional mimicry or physical interactions between CB1R and D2R in striatal cells (77) may therefore inhibit the striatopallidal pathway, leading to locomotor disinhibition, especially given the increased dopamine release by THC in the basal ganglia. However, a contribution of D1-MSN CB1R to the hyperlocomotor response to a low dose of THC cannot be fully excluded due to incomplete reduction of CB1R in D1+ cells in Drd1-CB1R-KO mice (22).

Because phosphorylation at S9 in the N-terminal tail of GSK-3β inhibits its substrate-binding domain (30), we used viral interference against GSK-3β^S9^ phosphorylation in Adora2a-Cre mice to demonstrate that GSK-3β inhibition is key to THC-induced psychomotor behavior via CB1R at D2-MSNs. Conversely, while ERK^1/2^/MAPK signaling modulates motor function in both MSN populations (78), p-ERK^1/2^/MAPK levels were unaltered in our conditions. Although several time points were not tested, this absence of activation fits with the hyperlocomotor phenotype of mice lacking ERK^1/2^/MAPK in D2-MSNs (78), suggesting that increased D2-MSN ERK^1/2^/MAPK signaling mediates hypolocomotion, whereas THC-induced hyperlocomotion requires D2-MSN GSK-3β signaling. These findings align with reports that deleting GSK-3β in D2R-expressing cells blunts psychostimulant-induced locomotion (79), further supporting a central role for GSK-3β in D2-MSNs in motor stimulation.

### Mechanism of Action of PREG and AEF0117 as a Therapeutic Strategy for THC-Induced Psychotic-Like States

We demonstrated that both PREG and AEF0117 inhibit GSK-3β–dependent THC-induced hyperlocomotion (Figure 5), strongly suggesting a biased modulation of CB1R-mediated β-arrestin 1/Akt/GSK-3β signaling. This is highly relevant to the endogenous mechanism of CB1-SSi, namely PREG (11) and the first-in-class clinical candidate, AEF0117 (21). Furthermore, our results support the involvement of H8/TM7 in the β-arrestin–biased signaling state of CB1R (37,80). Indeed, the allosteric site of PREG and AEF0117, encompassing TMH1, TMH7, and the intracellular helix H8 of CB1R, was validated using a single point mutation (p.E1.49G) in human CB1R, which disrupted their binding and failed to block THC-mediated suppression of cellular respiration in human embryonic kidney 293 cells (11,21). Although further investigations are needed to confirm a direct effect on β-arrestin 1–biased CB1R signaling, this unique mechanism highlights the therapeutic potential of AEF0117 (21) for treating cannabinoid-related psychotic-like conditions.

### Strengths and Limitations

A key strength of our study lies in its multitiered approach to unraveling the mechanisms of THC-induced hyperlocomotion. We combined high-throughput in vitro assays, pharmacological tools, and transfection strategies with genetic mouse models and striatal viral injections. Crucially, evaluating a signaling-specific inhibitor that reverses both the molecular and behavioral phenotypes of low-dose THC expands the therapeutic scope of AEF0117 to include cannabinoid-induced acute psychotic-like disorders. Our preclinical framework helps to consolidate several aspects of the validity criteria of this psychosis-like model (81), including face validity by behaviorally mimicking positive-like symptoms, construct validity through striatal cell type–specific CB1R signaling pathways (i.e., GSK-3β in D2-MSNs), and predictive validity via the discovery of AEF0117 as a prospective therapeutic.

Furthermore, we revealed that GSK-3β–dependent hyperlocomotion recruits a broad circuit encompassing the dorsal striatum, nucleus accumbens, and cerebellum. These findings support the anatomical relevance of the cerebello–basal ganglia network (82,83) and extend its functional involvement to acute cannabis-induced psychotic-like states. This network is particularly relevant to cannabinoid neurobiology, as its nodes display among the highest CB1R expression levels in both human and rodent brains (84). While chronic THC exposure downregulates CB1R (85,86), rapid downregulation can also occur early after an acute high-dose challenge (87). Although such changes have not been reported after an acute low dose, transient receptor internalization or downregulation remains a plausible mechanism contributing to the intracellular signaling alterations identified here. Conversely, unlike chronic paradigms (88,89), endocannabinoid tone is unlikely to be altered, as acute cannabis or THC administration does not affect endocannabinoid levels in human plasma (65) or mouse brain (90), respectively.

Several limitations warrant consideration. First, an acute THC challenge does not fully capture the complex, chronic dynamics linking cannabis use to psychosis. While heavy use increases the risk of psychotic episodes (91,92), translational modeling of chronic administration remains challenging (93), notably due to rapid tolerance (58) that blunts hyperlocomotor responses upon repeated low-dose exposure (86). Second, our study evaluated exclusively male mice, limiting the generalizability of our findings. This design was chosen based on a previously validated male mouse model demonstrating THC-induced hyperlocomotion and its rescue by PREG and AEF0117 (12,21). Although early-onset psychosis is more prevalent in men, partly due to higher rates of cannabis consumption (94), emerging evidence suggests that women may exhibit heightened vulnerability to cannabis-induced psychosis (95). Incorporating female cohorts in future studies will be essential to resolving potential sex-dependent mechanisms in the psychomimetic effects of cannabinoids.

### Conclusions

Our findings demonstrate that THC-induced psychotomimetic hyperlocomotion, at a dose that elevates striatal THC levels, requires CB1R located specifically on the membrane of striatopallidal neurons. Mechanistically, this effect is driven by the inhibition of striatal GSK-3β signaling activity through a β-arrestin 1–dependent CB1R signaling pathway. Beyond deciphering this circuit and pathway mechanism, our work reveals a key mode of action of CB1-SSi compounds and identifies AEF0117 as a potential therapeutic strategy for cannabinoid-induced psychotic-like states.

## Supplementary Material

Supplement Methods, Figures S1–S8, and Table S1

Key Resources Table
