# Supplementary material for: Psychotomimetic Cannabinoid Hyperlocomotion Involves GSK-3β Signaling at Striatopallidal Neurons in Male Mice
Source: Biol Psychiatry Glob Open Sci. 2026 Aug 28;6(6):100816. doi: 10.1016/j.bpsgos.2026.100816 (PMC13685958; doi:10.1016/j.bpsgos.2026.100816)
Supplement: Supplementary file 1 [file mmc1.pdf]

SUPPLEMENTARY INFORMATION

Psychotomimetic Cannabinoid Hyperlocomotion Involves GSK3β Signaling at Striatopallidal Neurons in Male Mice

Tomaselli *et al.*

Supplementary Methods

Subjects. Animals were maintained in group housing, and were single-housed in standard plastic rodent cages one week prior to the experiments. Cages and enrichment (cellulose nests and cardboard tunnels) were changed every two weeks. All mice were housed in the animal facility under controlled conditions of temperature (range 20-22°C) and humidity (range 55-60 %) with a constant 12-hour light/dark cycle (light on at 7 am) and *ad libitum* access to food (Standard Rodent Diet A04, SAFE, France) and water.

C57BL/6N mice (weighing 25-30 g) were purchased from Janvier Laboratories (France) and the following mouse lines were generated in our animal facility. β-Arrestin 1 constitutive KO (B6.129X1(Cg)-Arrb1^{tm1Jse}/J – Arrb1) mice were purchased from The Jaxon Laboratory (JAX stock #011131). β-Arrestin 1 (-/-) KO mice were obtained by breeding heterozygous male and female β-Arrestin 1 (-/+) mice. CB1-flox mice, generated and maintained as previously described (1) were used to generate conditional mouse lines and for the viral deletion experiment. Conditional CB1R mutant mice and their WT littermates were generated by a 3-step breeding scheme using the Cre-loxP system and maintained in our animal facility. To generate the Adora2a-CB1-KO line, CB1-flox mice were crossed with Adora2a-Cre mice (Tg(Adora2a-Cre)KG12633); in Adora2a-Cre mice, provided by MGI (Jackson Laboratory, USA), the Cre recombinase was placed under the control of the adenosine A2A receptor gene (Adora2A) regulatory sequences using BAC transgenesis. Similarly as described above, to generate Drd1-CB1-KO mice, CB1-flox mice were crossed with Drd1-Cre mice (Tg(Drd1-Cre)EY217), in which the Cre recombinase is inserted into the regulatory sequence of the dopamine receptor d1 gene (Drd1a33), provided by MGI (Jackson Laboratory, USA). WT controls for each of these 2 lines were CB1-flox littermates without Cre expression.

Drug Preparation and Administration. THC was purchased as dronabinol resinous oil or 10 mg/ml solution in 100% ethanol. The resin was dissolved at 50 mg/ml (w/v) in 100% ethanol. For *in vivo* experiments, ethanol solutions were solubilized in a solution of 4% ethanol, 4% cremophor, 92% saline (0.9% NaCl) and administered to mice by intraperitoneal (i.p.) injection in a volume of 10 ml/kg, at a dose of 0.3 mg/kg. For *in vitro* experiments, ethanol solutions were evaporated to dryness, then dissolved in DMSO and used at a concentration of 10 μM for cell

treatment. GDC-0084 ((5-(6,6-dimethyl-4-morpholino-8,9-dihydro-6H-[1,4]oxazino[4,3-e]-purin-2-yl)pyrimidin-2-amine) was dissolved in a solution of 0.2% methylcellulose, 0.2% Tween80 for oral administration (*p.o.*) via gavage at a volume of 5 ml/kg. D-amphetamine sulfate was dissolved in saline (0,9% NaCl) and injected i.p. at 5 mg/kg in a volume of 10 ml/kg. Haloperidol (Haldol®) was dissolved in saline (0,9% NaCl) and injected i.p. at 0.1 mg/kg in a volume of 10 ml/kg. Pregnenolone (3β-Hydroxy-5-pregnen-20-one, 5-Pregnen-3β-ol-20-one) was dissolved in a solution of 1% Tween80, 2% DMSO, 97% saline (0.9% NaCl) to be administered subcutaneously (s.c.) in a volume of 10 ml/kg, at a dose of 6 mg/kg. AEF0117 was provided by Aelis Farma SA and dissolved in a corn oil solution to be administered *p.o.* by gavage in a volume of 5 ml/kg at a dose of 15 μg/kg.

Experimental design and timeline. The effects of THC, either by itself or in combination with pre-treatment with GDC-0084, PREG, or AEF0117, on locomotion and CB1-mediated signaling pathways were investigated in C57BL/6N mice (see **Figures 1, 2,** and **5**). The timing of the 10-minute pre-treatment and the 6 mg/kg dose of i.p. pre-treatment with PREG were chosen based on previous data showing reversal of THC-induced hyperlocomotion in mice (2). The timing of pre-treatment with GDC-0084 or AEF0117 was determined based on prior pharmacokinetic studies. These studies showed that GDC-0084 and AEF0117 reached steady state (T max) levels in the brain after one and three hours, respectively, following oral administration in mice (3). Additionally, oral administration of AEF0117 at the dose of 15 μg/kg was able to reverse THC-induced hyperlocomotion in mice (3). To determine whether β-arrestin 1 was necessary for THC-induced hyperlocomotion, we examined the effect of THC on locomotion was conducted in β-arrestin 1 KO mice (see **Figures 3C** and **3D**). To determine the (sub)cellular localization of CB1R involved in THC-induced hyperlocomotion, the effects of THC on locomotion were studied in several conditional mutant mouse lines, including DN22-CB1-KI (4,5), GABA-CB1-KO (6,7), Drd1-CB1-KO and Adora2a-CB1-KO mice (1) (see **Figure 4 A-D**). THC-induced hyperlocomotion was measured 45 after intraperitoneal (i.p.) injection of THC, as previously described, in C57BL/6N mice (2). The dose effects of GDC-0084 on locomotion were assessed in C57BL/6N mice (see **Figure S2**). The effect of a 10-minute i.p. pre-treatment with haloperidol (0.1 mg/kg) on THC-induced hyperlocomotion was evaluated in C57BL/6N mice (see **Figure S5**). As shown in **Figure S5**, the

timing and dosage of the haloperidol treatment were adjusted so that the treatment itself did not affect locomotion.

Locomotion tests. To study the effects of THC on locomotion, mice were placed in the center of a transparent open field (45 x 35 x 30 cm) with a square-patterned floor (10 cm^2 each) under controlled light (110 lux). Locomotor activity was assessed by the number of crossed squares and rearings counted during 5 min by the experimenter. A locomotor index including horizontal and vertical locomotion (crosses + rearings) was calculated. Pre-treatment with PREG, AEF0117, GDC-0084 or their vehicle were performed in independent groups of mice. As described in the previous section, locomotion was measured 45 minutes after the injection of THC or its vehicle combined with a 60-minute pre-treatment with GDC0084 or its vehicle, with a 10-minute pre-treatment with PREG or its vehicle (2), or with a 3-hour pre-treatment with AEF0117 or its vehicle (3). To determine the dose of GDC-0084 that is ineffective on its own, the locomotor effects of different doses were assessed one hour later in the open field, as described above.

Amphetamine-induced locomotor increase was assessed in the Actimeter apparatus, which consists of a ventilated rack containing 8 individual actimetry cages (30 x 16 x 11 cm). Each cage was equipped with infrared sensors to detect locomotor activity, and infrared planes to detect rearing. Beam-breaks were recorded and a locomotor index (horizontal and vertical locomotion) was calculated. Amphetamine was administered after one hour of habituation in an actimetry cage, then locomotion was assessed for one hour period (test) as previously described (1).

Viral Vector and surgery procedures. AAVs were generated by PEI transfection of HEK293T cells and purified by iodixanol-gradient ultracentrifugation as previously described (8). Viral titers were $1.60*10^{11}$ for AAV-DIO-GSK3β, $6.7*10^{11}$ for AAV-DIO-GSK3β^{S9A} and 4.51*1011 for AAV-DIO-ctr, expressed as genomic copies (GC) x ml. Viral injections of AAV-DIO-GSK3β and AAV-DIO-GSK3β^{S9A} in the dorsal striatum of Adora2A-Cre mice, with respective AAV-DIO-Ctr, were performed with the following coordinates: AP +0,5 L ± 2,0, DV –3.0 as previously described (9). Animals were allowed to recover for at least four weeks before the beginning of biochemical or behavioral experiments.

Immunofluorescence and double fluorescent *in-situ* hybridization (FISH) assay. Endogenous peroxidases from the cryosections were inactivated with 3% H_2O_2 diluted in PBS-DEPC for 30 min at RT. Slices were incubated for 20 min at RT in 0.2 mM HCl-DEPC following by an acetylation step of 10 min with 0.1M triethanolamine-HCl (pH 8) and 0,25% acetic anhydride. Then, slices were hybridized overnight at 70 °C with sense or antisense probes. After that, sections were incubated 1 hour at RT with anti-DIG POD antibody (1:1500) followed by 10 min incubation with TSA Plus Cyanine 3 (1:100). Afterward, sections were permeabilized and blocked for 1 hour at RT incubation in blocking solution with 0.3% of triton. Primary antibody rabbit anti-HA (1:1000) was incubated over night at 4ºC in PBS+0.3% Triton. After some washes, sections were incubated for 2h at RT in PBS+0.3% Triton with the secondary antibody donkey anti-rabbit AF 488 (1:500). Lastly, nuclei were stained with DAPI (1:20000 in PBS). Finally, sections were mounted, dried, coversliped and imaged with a Leica SP5 multi-photon microscope. All DEPC solution had a concentration of 5% v/v DEPC.

Brain tissue sampling and protein quantitation. To preserve protein phosphorylation, brain areas were rapidly dissected on ice and placed in dry ice-cold Precellys tubes stored at -80°C prior to protein extraction and AlphaLISA assay, as previously described (10). Extraction of total proteins from mouse brain structures was performed in AlphaLISA SureFire Ultra lysis buffer supplemented with protease and phosphatase inhibitors using the Precellys-24 homogenizer. A two-cycle homogenization protocol (30 s at 5000 rpm) with a 10 s pause between the two cycles using CK14 ceramic beads was used. After homogenization, the samples were centrifuged three times at 10.000 rpm for 10 min at 4 °C, and then the supernatants were quantified. Total protein quantification was performed using a Direct Detect X spectrometer that analyses the amount of amine bonds (Merck Millipore) by mid-infrared spectroscopy. Protein samples were then stored at -80ºC until AlphaLISA analysis.

Alpha (Amplified Luminescent Proximity Homogeneous Assay) Analyses. 10 µl/well of diluted protein samples and 5 µl of acceptor mix (containing both antibodies and acceptor beads) were added in an OptiPlate-384 white opaque microplate. The plate was sealed with a transparent adhesive film and incubated at room temperature for one hour. 5 µl of donor mix (containing Donor

beads) was then added into the wells under subdued light (<100 lux). The plate was resealed and incubated at room temperature for one hour. The plate was then read on an Alpha technology-compatible plate reader. Samples were diluted in lysis buffer to the desired concentration as follows: 0.625 µg / 10 µl for p-Erk1/2$^{T202/Y204}$, total Erk1/2, and p-4EBP1$^{T37/46}$; 1.25 µg / 10 µl for p-GSK3β^{S9}, and p-RPS6$^{S240/244}$; 2.5 µg / 10 µl for total cofilin, p-Akt1/2/3^{T308}, p-Akt1/2/3^{S473} and p-MEK1$^{S218/222}$, and 5 µg / 10 µl for p-mTOR^{S2448}. The housekeeping protein cofilin was used as a loading control to ensure the reliability of the data in all Alpha assays and to determine if the differences in the phosphorylated targets were solely the result of THC treatment (10).

Cell cultures. One day before the transfection, STH*dh*$^{Q7/Q7}$ cells were plated in a 96 well plate at 2x104 cells per well in 100µL of complete medium (DMEM, 10% FBS not inactivated, 1% penicillin/streptomycin, 1% G418, stock 40 mg/mL). Cells were incubated in a humidified atmosphere at 33°C with 5% CO2. Then STH*dh*$^{Q7/Q7}$ cells were transfected with Lipofectamine 2000 reagent in OptiMEM medium according to the manufacturer's instructions at a final ratio of 120 µg cDNA / 0.4 µl Lipofectamine per well with *ARRB1* gene or pcDNA3.1(+). 48h post-transfection, the cells were treating with THC or its vehicle and allowed to incubate for 30 minutes. 24h post-transfection, cells were deprived of FBS, penicillin, streptomycin, and G418 and maintained in 100 µl of DMEM per well. After 24 h of deprivation, the culture medium was removed and changed with fresh DMEM (50 µL). The cells were then treated by adding 150 µL of THC solution (40 µM in DMEM, final concentration 10 µM) or its vehicle (DMEM, DMSO 0.2%; final concentration of DMSO, 1.05%). After 30 minutes of incubation at 33°C, the culture medium was removed, and the cells were lysed with 100µL of AlphaLISA lysis buffer supplemented with phosphatase and protease inhibitors (1% and 0.5%, v/v respectively). The plate was shaken on a plate shaker (350 rpm) for 10 min at room temperature.

Gene expression analyses

RNA extraction. Total RNA was extracted using a standard chloroform/isopropanol protocol and purified by incubation with AmbionTM DNAse I - RNAse free (11). Transcript-specific primers were generated using Primer Express software based on GenBank sequence information, verified by NCBI BLAST search, and custom synthesized. Sequences of all primer pairs are listed in

Supplementary Table 1. Purity and concentration of RNA samples were determined from OD260/280 readings using the ND1000 UV spectrophotometer (Fisher Scientific), and RNA integrity was determined by capillary electrophoresis using the RNA 6000 Nano Lab-on-a-Chip kit run on the Bioanalyzer 2100. cDNA was synthesized from 2 µg of RNA using RevertAid Premium Reverse Transcriptase (Fermentas) with a mixture of random primers (Fermentas) and oligo(dT)18 primers (Fermentas).

Real-time quantitative PCR (RT-qPCR). PCR conditions and LightCycler® 480 SYBR Green I Master mix were used for a 10 µl reaction with transcript-specific primers (0.6 µM) and 2 µl cDNA, corresponding to 4 ng total RNA input. An initial denaturation step at 95 °C for 5 min was followed by 45 cycles of 95°C for 15 sec, 61°C for 15 sec. The relative gene expression levels of the transcripts were analyzed by the comparative Cq method (2-ΔΔCT) implemented in GEASE (Gene Expression Analysis Software Environment) IT tool developed at the Neurocentre Magendie (Bordeaux), using two reference genes for normalization (Eef1a1, eukaryotic translation elongation factor 1 alpha 1 and Nono, non-POU domain containing octamer binding). Each primer set was tested for the absence of primer-dimer artifacts and multiple products by melting curve analysis and gel electrophoresis. The amplification efficiency of each set was determined by repeated dilution series of a cDNA mixture. Only primers with an efficiency of ~2 were used.

Droplet digital PCR (ddPCR). The ddPCR mixtures were prepared in a final volume of 20 µl containing the required QX200 ddPCR EvaGreen Supermix with a final concentration of 150 nM of each primer sets and 2 µl cDNA equivalent to 4 ng total RNA input. Each ddPCR assay mixture (20 µl) was loaded into a disposable droplet generator cartridge (Bio-Rad). Then, 70 µl of droplet generation oil was distributed into each of the eight oil wells of the cartridge. The cartridge was then placed in the QX200 droplet generator. When droplet generation was complete, the droplets were transferred to a 96-well PCR plate, heat-sealed with foil in a PX1 PCR Plate Sealer and amplified using a Mastercycler Nexus Gradient Thermal Cycler. Thermal cycling conditions for EvaGreen assays were as follows: 95°C for 5 min, followed by 45 cycles of 95°C for 30 s and 61°C for 1 min, followed by 4°C for 5 min and a final inactivation step at 90°C for 5 min. A no template control and a negative control were included in the assay for each reverse transcription reaction. After thermal cycling the sealed plate was placed in the QX200 Droplet Reader for data acquisition. The resulting

data were analyzed using QuantaSoft software. For each set of primers, thresholds of fluorescence amplitude were manually set to distinguish between positive and negative droplets. Only the reactions with more than 15,000 valid droplets were used for analysis.

Plasma and brain sampling. The brain was quickly removed and brain areas, including the dorsal striatum, were harvested on ice. Blood samples were collected in EDTA-coated tubes. After centrifugation at 500g for 20 min under refrigeration (4 ± 2 °C), the plasma supernatant was removed. All samples were snap frozen and stored at -80 °C until mass spectral analysis.

THC assay. THC assay was performed using an isotope dilution method (with internal standard deuterated THC analog, THC-d3) combined with liquid chromatography (LC)-chemical ionization-tandem mass spectrometry (MS/MS) analysis as previously described (3,12,13). Mass spectral analyses were performed on a TSQ Quantum Access triple quadrupole (Thermo-Finnigan) instrument equipped with an APCI (atmospheric pressure chemical ionization) source operating in positive ion mode, in conjunction with a Surveyor LC Pump Plus (Supelco C18 Discovery Analytical column) and cooled autosampler. Briefly, samples were homogenized with spiked THC-d3 and the lipid fraction was extracted by using a liquid-liquid extraction with chloroform/methanol/Tris-HCL 50 mM pH 7.5 (2:1:1, v/v) and purified by using a solid-phase extraction (SPE C18; Agilent) with cyclohexane/ethyl acetate (1:1, v/v). The samples were then subjected to LC-APCI-MS/MS in combination with quality control samples to assess between-run and within-run accuracy and precision (CV < 20%) and calibration curve samples (linearity > 0.99) to quantify THC. The mass spectrometer was operated in selected reaction monitoring (SRM) mode to enhance sensitivity, and the concentration of THC was calculated by linear regression of the peak area corresponding to the diagnostic fragment ion (m/z) with the highest intensity.

Statistical Methods. Statistical analyses were performed using GraphPad Prism 7.05 software. Normality was assessed with the D'Agostino & Pearson or Shapiro-Wilk Normality test. A Student's t-test was used for pairwise comparisons, and one-way or two-way analyses of variance (ANOVAs) were used for multiple group comparisons. Post hoc analyses using Sidak's multiple comparison tests were performed as appropriate when the ANOVA was significant. Outliers were identified using Grubb's test and then removed. The significance level was set at 0.05.

References

1. Mariani Y, Covelo A, Rodrigues RS, Julio-Kalajzić F, Pagano Zottola AC, Lavanco G, *et al.* (2023): Striatopallidal cannabinoid type-1 receptors mediate amphetamine-induced sensitization. *Curr Biol* 33: 5011-5022.e6.

2. Busquets-Garcia A, Soria-Gómez E, Redon B, Mackenbach Y, Vallée M, Chaouloff F, *et al.* (2017): Pregnenolone blocks cannabinoid-induced acute psychotic-like states in mice [no. 11]. *Mol Psychiatry* 22: 1594–1603.

3. Haney M, Vallée M, Fabre S, Collins Reed S, Zanese M, Campistron G, *et al.* (2023): Signaling-specific inhibition of the CB1 receptor for cannabis use disorder: phase 1 and phase 2a randomized trials. *Nat Med* 29: 1487–1499.

4. Pagano Zottola AC, Soria-Gomez E, Bonilla-del-Río I, Muguruza C, Terral G, Robin LM, *et al.* (2020): *A New Mutant Mouse Model Lacking Mitochondrial-Associated CB $_1$ Receptor*. Neuroscience. https://doi.org/10.1101/2020.03.30.009472

5. Soria-Gomez E, Pagano Zottola AC, Mariani Y, Desprez T, Barresi M, Bonilla-del Río I, *et al.* (2021): Subcellular specificity of cannabinoid effects in striatonigral circuits [no. 9]. *Neuron* 109: 1513-1526.e11.

6. Bellocchio L, Lafenêtre P, Cannich A, Cota D, Puente N, Grandes P, *et al.* (2010): Bimodal control of stimulated food intake by the endocannabinoid system [no. 3]. *Nat Neurosci* 13: 281–283.

7. Monory K, Blaudzun H, Massa F, Kaiser N, Lemberger T, Schütz G, *et al.* (2007): Genetic Dissection of Behavioural and Autonomic Effects of Δ9-Tetrahydrocannabinol in Mice [no. 10] ((E. Nestler, editor)). *PLoS Biol* 5: e269.

8. Hebert-Chatelain E, Desprez T, Serrat R, Bellocchio L, Soria-Gomez E, Busquets-Garcia A, *et al.* (2016): A cannabinoid link between mitochondria and memory [no. 7630]. *Nature* 539: 555–559.

9. Bellocchio L, Ruiz-Calvo A, Chiarlone A, Cabanas M, Resel E, Cazalets J-R, *et al.* (2016): Sustained Gq-Protein Signaling Disrupts Striatal Circuits via JNK. *J Neurosci* 36: 10611–10624.

10. Zanese M, Tomaselli G, Roullot-Lacarrière V, Moreau M, Bellocchio L, Grel A, *et al.* (2020): Alpha technology: A powerful tool to detect mouse brain intracellular signaling events. *Journal of Neuroscience Methods* 332: 108543.

11. Chomczynski P, Sacchi N (1987): Single-step method of RNA isolation by acid guanidinium thiocyanate-phenol-chloroform extraction [no. 1]. *Anal Biochem* 162: 156–159.

12. Kearney-Ramos T, Herrmann ES, Belluomo I, Matias I, Vallée M, Monlezun S, *et al.* (2022): The Relationship Between Circulating Endogenous Cannabinoids and the Effects of Smoked Cannabis. *Cannabis Cannabinoid Res*. https://doi.org/10.1089/can.2021.0185

13. Matias I, Belluomo I, Raux P-L, Vallée M (2023): Applied Clinical Tandem Mass Spectrometry-Based Quantification Methods for Lipid-Derived Biomarkers, Steroids and Cannabinoids: Fit-for-Purpose Validation Methods. *Biomolecules* 13: 383.

Supplementary Figures

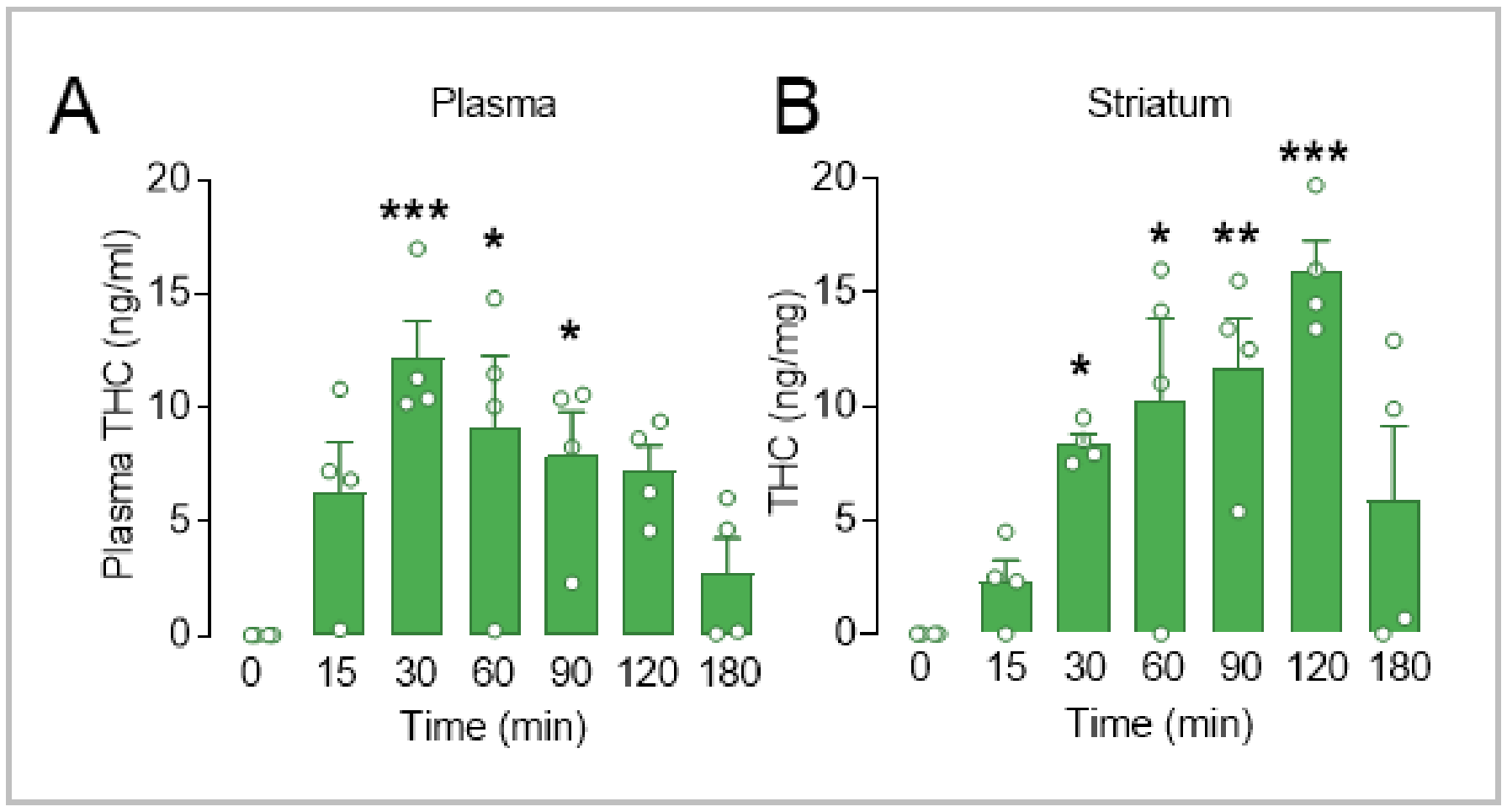

Figure S1. THC injection increased THC levels in the plasma and striatum of mice over time.

(**A**) Plasma THC levels peaked 30 min after i.p. injection of THC at 0.3 mg/kg and remained elevated for 90 min (n = 4 per group) ($F(6,21)=4.7$, $p<0.01$; T0 vs T15 : $p=0.11$, T0 vs T30 : ***$p<0.001$, T0 vs T60 : *$p<0.02$, T0 vs T90 : *$p<0.05$, T0 vs T120 : $p=0.056$, T0 vs T180 : $p=0.79$). (**B**) Striatal THC levels increased from 30 to 120 min after i.p. injection of THC at 0.3 mg/kg (n = 4 per group) ($F(6,21)=6.8$, $p<0.001$; T0 vs T15 : $p=0.92$, T0 vs T30 : *$p<0.05$, T0 vs T60 : *$p<0.02$, T0 vs T90 : **$p<0.01$, T0 vs T120 : ***$p<0.001$, T0 vs T180 : $p=0.24$). Data are mean ± SEM of single values. All statistical values are from a one-way ANOVA, followed by a Dunnett's multiple comparison test.

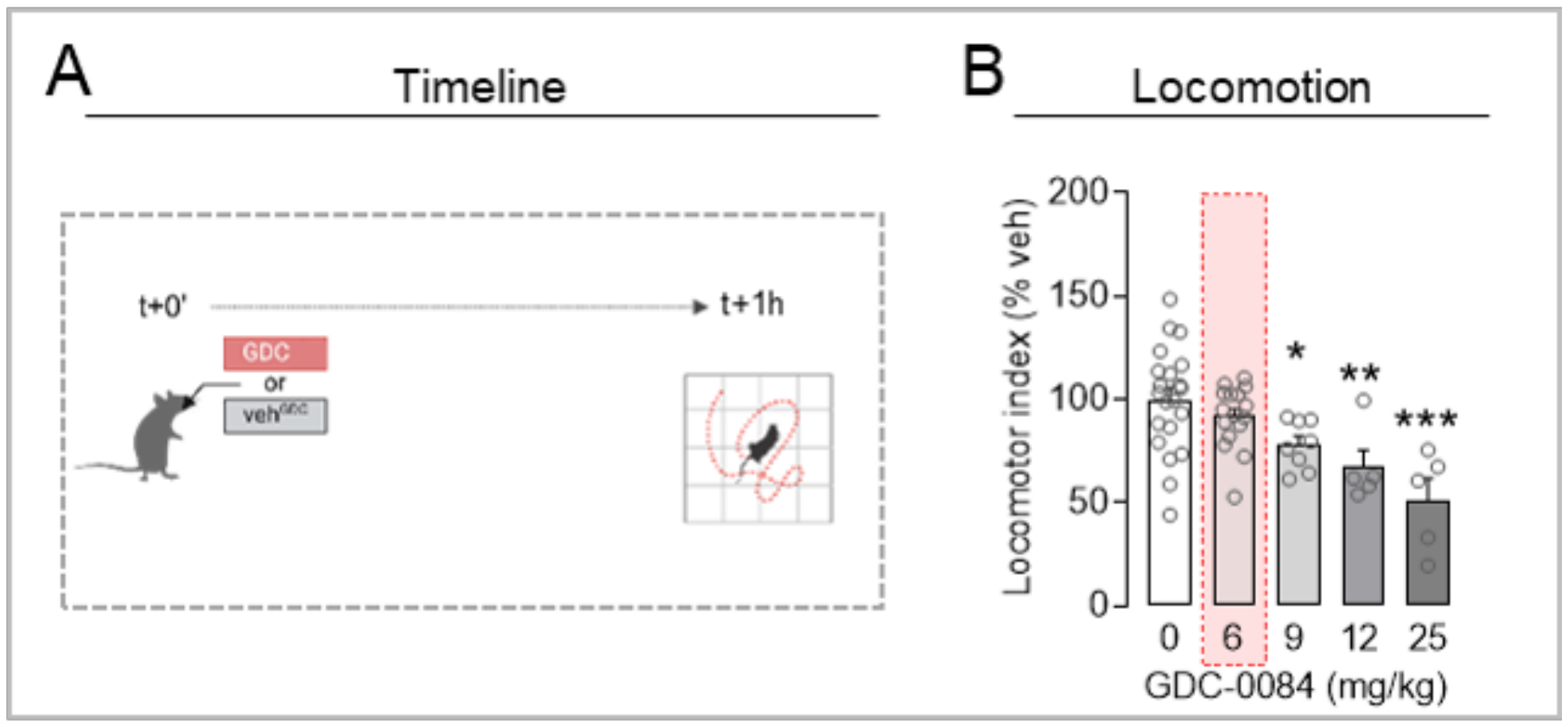

Figure S2. Dose-effect of GDC-0084 on locomotion in C57Bl/6N mice. (**A**). Locomotion is measured in an open-field 1 h after oral administration of GDC-0084 (at 6, 9, 12 or 25 mg/kg) (GDC) or vehicle (veh^{GDC}). (**B**). The locomotor index including horizontal and vertical locomotion (crosses + rearings) is expressed as a percentage of the vehicle. The 6 mg/kg dose was ineffective on locomotion and was therefore chosen to study the effect of GDC0084 on THC-induced locomotion (see **Figure 2**) (n = 5-21 per group) ($F(4,50)=7.6$, $p<0.001$; veh vs 6 mg/kg : $p=0.65$, veh vs 9 mg/kg : *$p<0.05$, veh vs 12 mg/kg : **$p<0.01$, veh vs 25 mg/kg : ***$p<0.001$). Data are mean ± SEM of single values. Statistical values are from a one-way ANOVA, followed by a Dunnett's multiple comparison test.

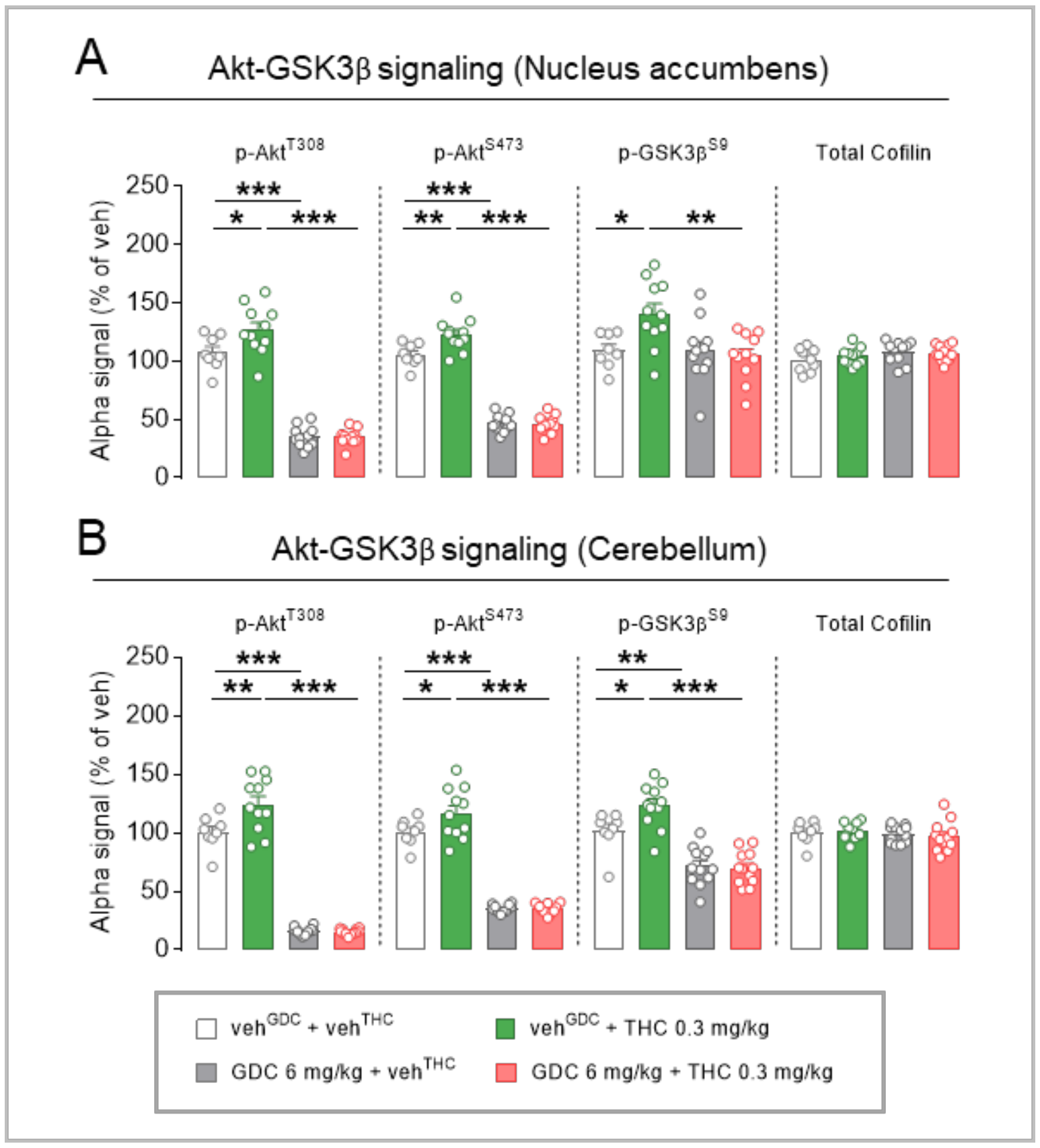

Figure S3. GDC-0084 blocks the effects of THC on Akt-GSK3β pathway activity in mouse brain tissue. Effect of GDC-0084 (6 mg/kg) on the phosphorylation of Akt (Thr308 and Ser473) and GSK3β (Ser9), and total cofilin in the nucleus accumbens (**A**) and cerebellum (**B**) of C57BL/6N mice treated with THC (0.3 mg/kg) or veh^{THC} (n = 9-12 per group) (Nucleus accumbens: p-Akt^{T308} : $F(1,39)=6$, $p<0.05$; veh^{veh} vs veh^{THC} : *$p<0.05$, veh^{veh} vs veh^{GDC} : ***$p<0.001$, veh^{THC} vs GDC^{THC} : ***$p<0.001$; p-Akt^{S473} : $F(1,39)=7.9$, $p<0.01$; veh^{veh} vs veh^{THC} : **$p<0.01$, veh^{veh} vs veh^{GDC} : ***$p<0.001$, veh^{THC} vs GDC^{THC} : ***$p<0.001$, p-$GSK3\beta^{S9}$: $F(1,37)=5.6$, $p<0.05$; veh^{veh} vs veh^{THC} : *$p<0.05$, veh^{veh} vs veh^{GDC} : $p=0.99$; veh^{THC} vs GDC^{THC} : **$p<0.01$; total cofilin : $F(1,39)=0.76$, $p=0.38$; Cerebellum: p-Akt^{T308} : $F(1,39)=8.7$, $p<0.01$; veh^{veh} vs veh^{THC} : **$p<0.01$, veh^{veh} vs veh^{GDC} : ***$p<0.001$, veh^{THC} vs GDC^{THC} : ***$p<0.001$; p-Akt^{S473} : $F(1,39)=4.8$, $p<0.05$; veh^{veh} vs veh^{THC} : *$p<0.05$, veh^{veh} vs veh^{GDC} : ***$p<0.001$, veh^{THC} vs GDC^{THC} : ***$p<0.001$, p-$GSK3\beta^{S9}$: $F(1,39)=5.3$, $p<0.05$; veh^{veh} vs veh^{THC} : *$p<0.05$, veh^{veh} vs veh^{GDC} : **$p<0.01$; veh^{THC} vs GDC^{THC} : ***$p<0.001$; total cofilin : $F(1,39)=0.26$, $p=0.61$). All assays were performed on the same tissue sample. Alpha signal is expressed as a percentage of veh^{THC}. Data are mean ± SEM of single values. All statistical values are from a two-way ANOVA, followed by a Sidak's multiple comparison test.

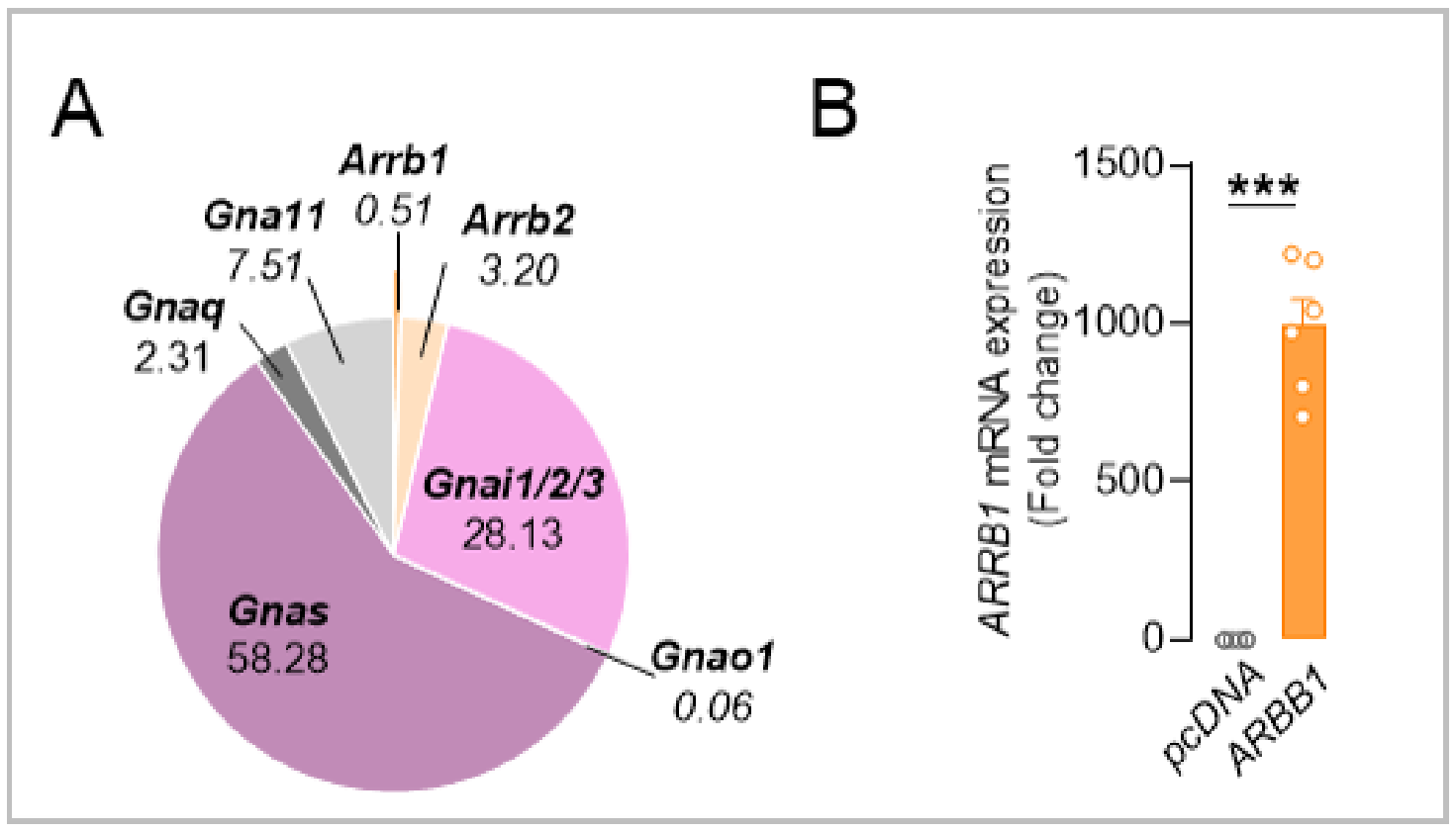

Figure S4. PCR analysis of β-arrestin and G-protein encoding genes in STH*dh*$^{Q7/Q7}$ cells. (**A**) Relative percentage of mRNA expression of β-arrestin (*Arrb1* and *Arrb2*) and G-protein (*Gnai1/2/3*, Gnao, *Gnaq*, *Gna11 and Gnas*) encoding genes measured by droplet digital PCR (ddPCR) in STH*dh*$^{Q7/Q7}$ cells. (**B**) *ARRB1* mRNA expression measured by real-time quantitative PCR (RT-qPCR) in STH*dh*$^{Q7/Q7}$ cells after 48 h post-transfection with human *ARRB1* encoding gene or with empty pcDNA (n = 5-7 per group) (Two-tailed unpaired t-test, *ARRB1* vs pcDNA : t=20, df=10, ***p<0.001). Data are mean ± SEM of single values.

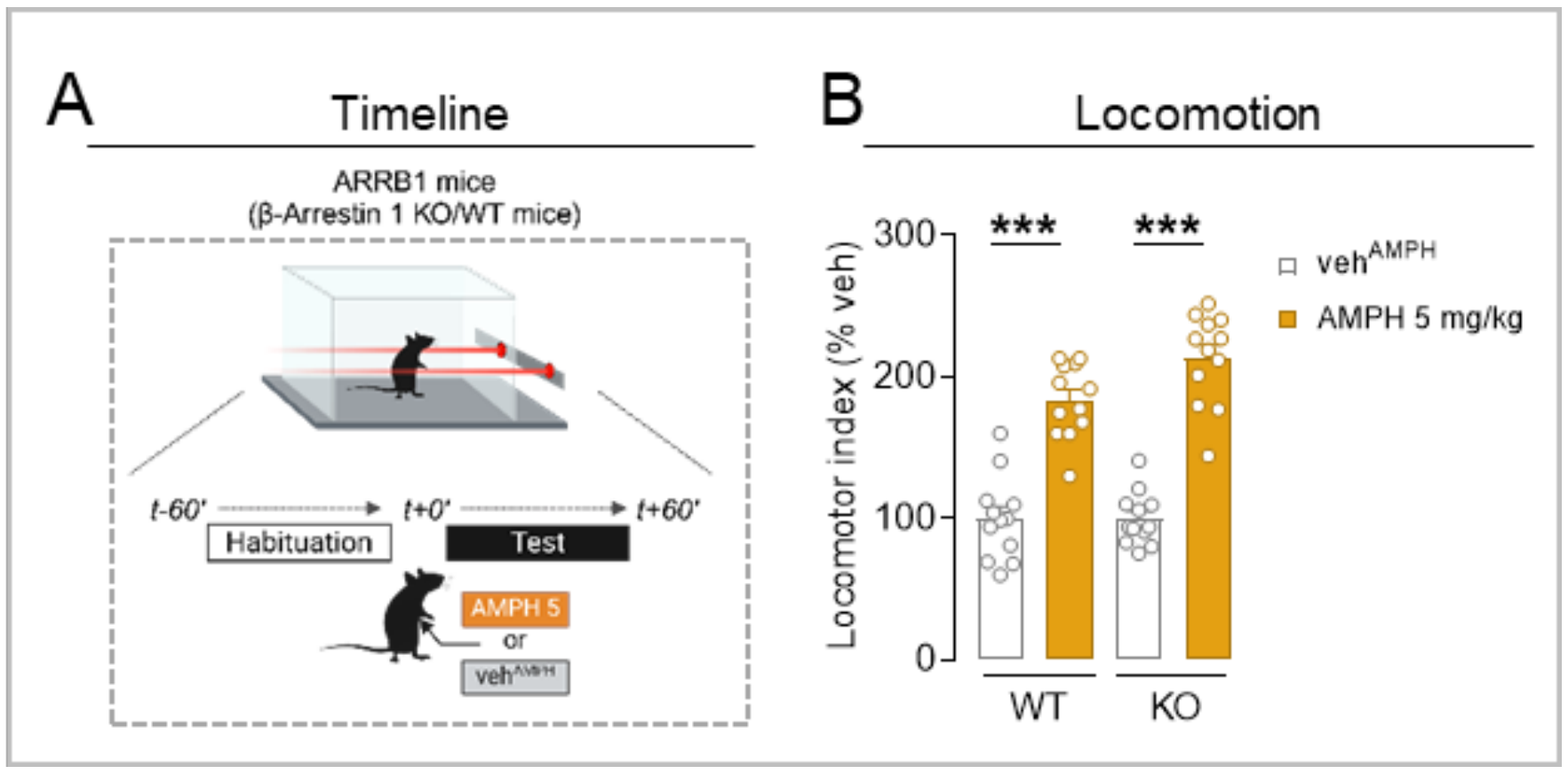

Figure S5. Amphetamine-induced hyperlocomotion in β-arrestin 1 knock-out (KO) mice. (**A**) Timeline of the locomotor activity procedure in β-arrestin 1 knock-out (KO) mice and wild-type (WT) littermates in the actimetry cages. Amphetamine (5 mg/kg) (AMPH 5) or vehicle (veh^{AMPH}) was administered i.p. after 1 h of habituation in the actimetry cages, then locomotion was assessed for 1 h (test period). (**B**) Effect of amphetamine on locomotor activity in WT and KO mice during the test period (n = 12 per group) ($F(1,44)=159$, $p<0.001$; WT^{veh} vs WT^{AMPH} : ***$p<0.001$, VEH^{veh} vs KO^{VEH} : $p>0.99$, KO^{veh} vs KO^{AMPH} : ***$p<0.001$, WT^{AMPH} vs KO^{AMPH} : $p=0.057$). The locomotor index, including horizontal and vertical locomotion, is expressed as a percentage of veh^{AMPH}. Data are mean ± SEM of single values. Statistical values are from a two-way ANOVA, followed by a Sidak's multiple comparison test.

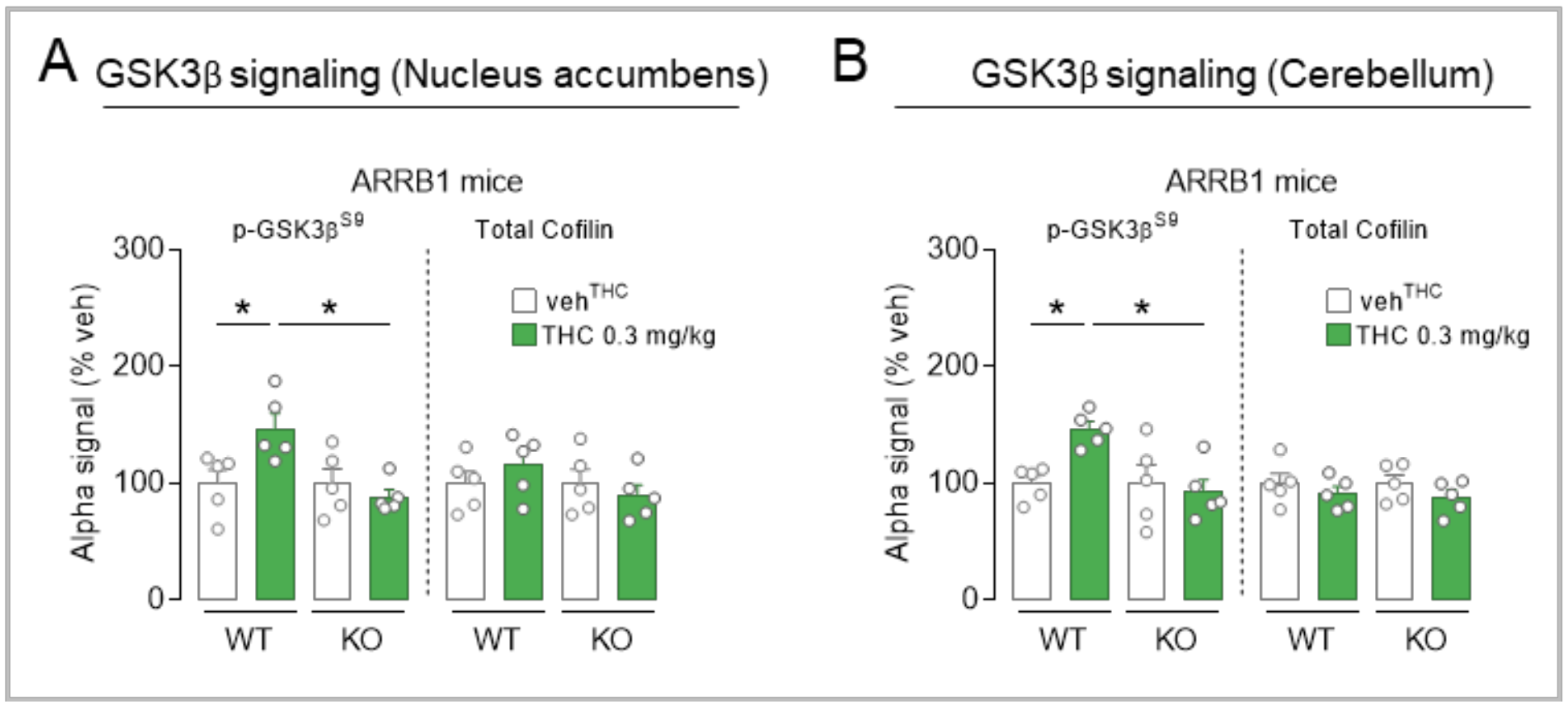

Figure S6. β-arrestin 1 is required for THC effects on GSK3β phosphorylation in mouse brain tissue. Effect of THC on the phosphorylation of GSK3β (Ser9) and total cofilin in the nucleus accumbens (**A**) and cerebellum (**B**) of WT and KO mice (n = 5-6 per group) (Nucleus accumbens : p-GSK3β^{S9} : F(1,16)=7.05, p<0.02; WT^{veh} vs WT ^{THC} : *p<0.05, KO^{veh} vs KO^{THC} : p=0.97, WT ^{THC} vs KO^{THC} : *p<0.02; total cofilin : F(1,16)=1.45, p=0.24; Cerebellum : p-GSK3β^{S9} : F(1,16)=6.47, p<0.05; WT^{veh} vs WT ^{THC} : *p<0.05, KO^{veh} vs KO^{THC} : p=0.99, WT ^{THC} vs KO^{THC} : *p<0.02; total cofilin : F(1,16)=0.05, p=0.82). Alpha signal is expressed as a percentage of veh^{THC}. Data are mean ± SEM of single values. All statistical values are from a two-way ANOVA, followed by a Sidak's multiple comparison test.

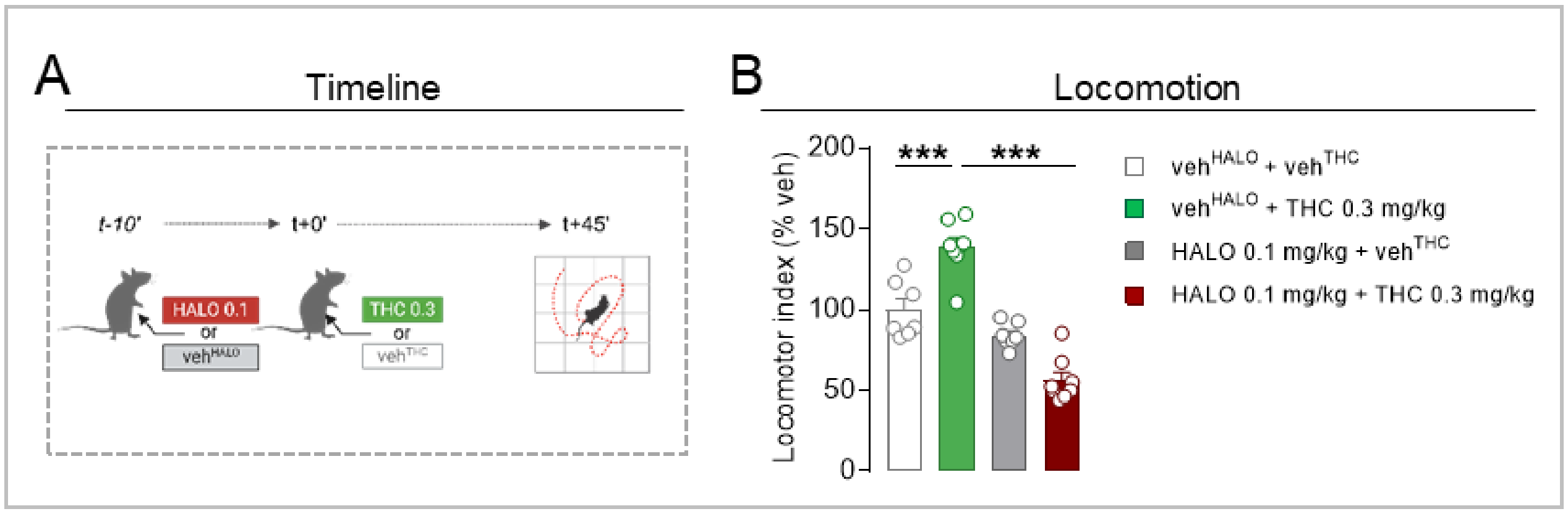

Figure S7. Haloperidol blocks the effects of THC on locomotion in C57Bl/6N mice. (**A**) Timeline of locomotor activity measurement in an open-field 45 min after i.p. injection of THC (0.3 mg/kg) or vehicle (veh^{THC}) in mice with a 10-minute pretreatment with i.p. injection of haloperidol (HALO) at 0.1 mg/kg or vehicle (veh^{HALO}). (**B**) Effect of haloperidol on locomotion in mice treated with THC or vehicle (veh^{THC}) (n = 12 per group) ($F(1,26)=39.3$, $p<0.001$; veh^{veh} vs veh^{THC} : ***$p<0.001$, veh^{veh} vs $HALO^{VEH}$: $p=0.27$, veh^{THC} vs $HALO^{THC}$: ***$p<0.001$, $HALO^{VEH}$ vs $HALO^{THC}$: **$p<0.01$). The locomotor index including horizontal and vertical locomotion (crosses + rearings) is expressed as a percentage of the vehicle. Data are mean ± SEM of single values. Statistical values are from a two-way ANOVA, followed by a Sidak's multiple comparison test.

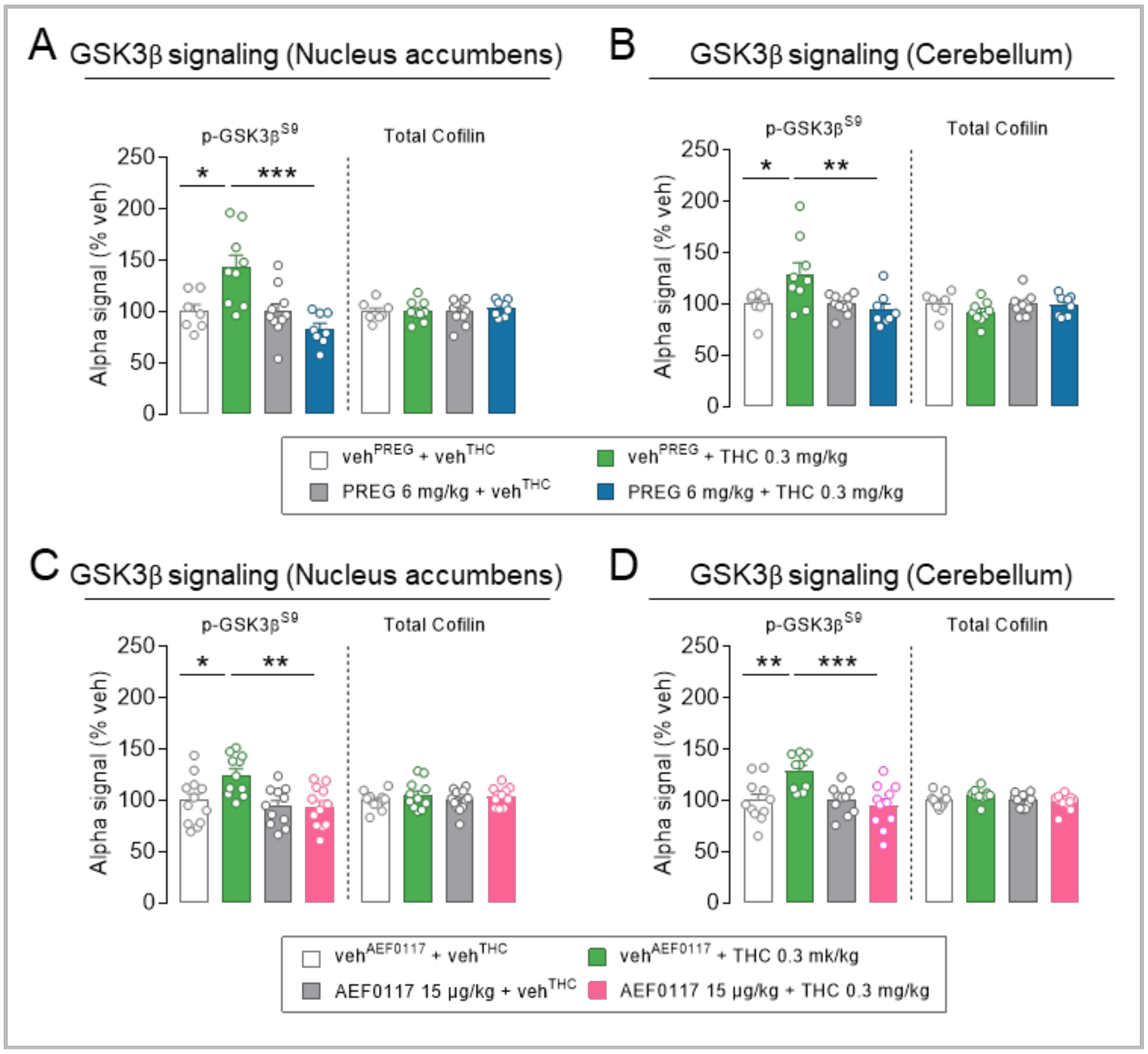

Figure S8. PREG and AEF0117 reverse CB1R-mediated effects of THC on Akt-GSK3β pathway activity in mouse brain tissue. (**A-B**). Effect of PREG on p-GSK3β^{S9} in the nucleus accumbens (**A**) and cerebellum (**B**) of C57BL/6N mice treated with THC or veh^{THC} (n = 8-10 per group) (Nucleus accumbens: p-GSK3β^{S9} : $F(1,30)=11$, $p<0.01$; veh^{veh} vs veh^{THC} : *$p<0.02$, veh^{veh} vs PREG^{VEH} $p=0.99$, veh^{THC} vs PREG^{THC} : ***$p<0.001$, PREG^{VEH} vs PREG^{THC} : $p=0.69$; total cofilin : $F(1,30)=0.17$, $p=0.68$; Cerebellum : GSK3β^{S9} : $F(1,30)=6.8$, $p<0.02$; veh^{veh} vs veh^{THC} : *$p<0.05$, veh^{veh} vs PREG^{VEH} : $p=0.99$, veh^{THC} vs PREG^{THC} : **$p<0.01$, PREG^{VEH} vs PREG^{THC} : $p=0.97$; total cofilin : $F(1,30)=0.89$, $p=0.35$). (**C-D**) Effect of AEF0117 on p-GSK3β^{S9} in the nucleus accumbens (**C**) and cerebellum (**D**) of C57BL/6N mice treated with THC or veh^{THC} (n = 9-12 per group) (Nucleus accumbens : p-GSK3β^{S9} : $F(1,38)=7$, $p<0.02$; veh^{veh} vs veh^{THC} : *$p<0.05$, veh^{veh} vs AEF^{VEH} : $p=0.99$, veh^{THC} vs AEF^{THC} : **$p<0.01$, AEF^{VEH} vs AEF^{THC} : $p=0.98$; total cofilin : $F(1,38)=0.47$, $p=0.49$; Cerebellum : GSK3β^{S9} : $F(1,38)=8.8$, $p<0.01$; veh^{veh} vs veh^{THC} : **$p<0.01$, veh^{veh} vs AEF^{VEH} : $p=0.99$, veh^{THC} vs AEF^{THC} : ***$p<0.001$, AEF^{VEH} vs AEF^{THC} : $p=0.99$; total cofilin : $F(1,38)=2.6$, $p=0.11$). For all AlphaLisa analyses, there was no difference in total cofilin between groups. p-GSK3β^{S9} and total cofilin assays were performed on the same tissue sample. Data are expressed as a percentage of veh^{THC}. Data are mean ± SEM of single values. All statistical values are from a two-way ANOVA, followed by a Sidak's multiple comparison test.

Supplementary Tables

Table S1. Primer sequences.

Gene name	GenBank ID°	Forward (5'-3')	Reverse (5'-3')
**Arrb1**	NM_177231	CCCCATGTGTGAAGGGCTAGTA	TCACCCTCATATCCCTTTCAGTAGT
**Arrb2**	BC016642	CCGCTATGGCCGAGAAGAC	CTGGTAGGTGGCGATGAACA
**Gnai1**	NM_010305	TCGTGCCATTGAAACAAAATCA	TGGGAAGGCGAGTCAGCTT
**Gnai2**	NM_008138	GCCGGGAGTAGCCATGGTA	CCCCAGAACAAGACAACGGTTA
**Gnai3**	NM_010306	GCTGTGGCTCGGATCCATT	CAGTTCTGACCATCAACCTTCTTTC
**Gnao1A**	NM_010308	AAGCCCACGGGTCTTTGTAA	AGGCTGGCAGTGTCAGGTGT
**Gnao1B**	NM_001113384	TTCTGGGCCTCCCGGTAA	GAGGTGGAGCCTCAGGACTG
**Gnaq**	NM_008139	CTTCTTAGGAGCCTGCGTATATTGT	GTTCCAGGTGTTCTTTAAATATCGC
**Gna11**	NM_010301	CCACCTTCCCACACTAGGCTC	TGTAGTGTATGGGATGGCACAGAT
**Gnas**	NM_201618	GTGACCCGGGCCAAGTACT	TGGCGCCCATCTCCACTA
**Reference genes**			
**Eef1a1**	NM_025380	TGAAAATCAGTGGCTCAACTTTAAA	ACACGAAAAAAGTAAACAGCCAGA
**Nono**	NM_023144	CTGTCTGGTGCATTCCTGAACTAT	AGCTCTGAGTTCATTTTCCCATG
